# Space Debris Removal: Learning to Cooperate and the Price of Anarchy

**DOI:** 10.3389/frobt.2018.00054

**Published:** 2018-06-04

**Authors:** Richard Klima, Daan Bloembergen, Rahul Savani, Karl Tuyls, Alexander Wittig, Andrei Sapera, Dario Izzo

**Affiliations:** ^1^Department of Computer Science, University of Liverpool, Liverpool, United Kingdom; ^2^Centrum Wiskunde & Informatica, Amsterdam, Netherlands; ^3^School of Engineering Sciences, University of Southampton, Southampton, United Kingdom; ^4^Advanced Concepts Team, European Space Agency, Noordwijk, Netherlands

**Keywords:** space debris, active debris removal, tragedy of the commons, price of anarchy, markov decision process

## Abstract

In this paper we study space debris removal from a game-theoretic perspective. In particular we focus on the question whether and how self-interested agents can cooperate in this dilemma, which resembles a tragedy of the commons scenario. We compare centralised and decentralised solutions and the corresponding price of anarchy, which measures the extent to which competition approximates cooperation. In addition we investigate whether agents can learn optimal strategies by reinforcement learning. To this end, we improve on an existing high fidelity orbital simulator, and use this simulator to obtain a computationally efficient surrogate model that can be used for our subsequent game-theoretic analysis. We study both single- and multi-agent approaches using stochastic (Markov) games and reinforcement learning. The main finding is that the cost of a decentralised, competitive solution can be significant, which should be taken into consideration when forming debris removal strategies.

## 1. Introduction

The Earth’s orbits are becoming increasingly cluttered with so-called *space debris*, made up by inactive or defunct satellites, rocket bodies, or other parts of spacecraft that have been left behind.^1^ This is particularly true for the Low Earth Orbit (LEO, defined as the region of space around Earth within an altitude of 160 to 2,000 km) environment, in which a large number of active satellites operate. This causes a substantial operational risk, ranging from the need to perform evasive manoeuvres to defects or even obliteration of spacecraft due to collisions with pieces of debris, which at orbital speeds of approximately 7.5 km/s can cause considerable damage.

To counter this risk, mitigation strategies are now implemented in newly launched satellites such as end-of-life de-orbiting or graveyard orbits (Inter-Agency Space Debris Coordination Committee and others, 2002; Klinkrad et al., 2004). However, researchers doubt that these measures, even if applied to all newly launched spacecraft, are sufficient to prevent a potential exponential build-up of debris (Liou and Johnson, 2006; Lewis et al., 2012). *Active space debris removal*, though very costly, may offer a solution (Klinkrad and Johnson, 2009; Izzo et al., 2015).

An active debris removal mission, if successful, has a positive effect (risk reduction) for all satellites in the same orbital band. This may lead to a dilemma: each stakeholder has an incentive to delay its actions and wait for others to respond. This makes the space debris removal setting an interesting strategic dilemma. As all actors share the same environment, actions by one have a potential immediate and future impact on all others. This gives rise to a social dilemma in which the benefits of individual investment are shared by all while the costs are not. This encourages free-riders, who reap the benefits without paying the costs. However, if all involved parties reason this way, the resulting inaction may prove to be far worse for all involved. This is known in the game theory literature as the tragedy of the commons. This dilemma is often studied as a one-shot interaction in which players (the actors) choose their strategy simultaneously and without communication. Most real scenarios however do not follow this abstract set-up, but are rather played out over multiple rounds of interactions, in which previous outcomes may influence future strategy choices. In this paper we analyse this more realistic scenario by modelling the space debris removal dilemma as a stochastic game, in which players decide on their strategy at multiple time points, and the combination of their choices influences the future development of the space debris environment.

The objective of this work is to model this strategic dilemma, understand its consequences and analyse various centralised and decentralised solution methods (including reinforcement learning). This requires us to provide a way to estimate the effect of certain actions on the space environment and assets held by the actors. To this end, we build on and extend a previously developed full scale simulator (Klima et al., 2016a) to include smaller debris and more realistic future launch scenarios and debris mitigation strategies. Using data collected from the full scale simulator we then build and validate an approximate surrogate model that can subsequently be used to efficiently test the effects of various debris removal strategies without requiring the computing power needed to run the full scale simulator. Additionally, the surrogate model can be used as a basis for further game theoretic analysis through the definition of a *stochastic game*. We provide illustrative test cases that demonstrate the potential use of our surrogate model for space actors and policy makers.

The contributions of this paper to the state of the art in the field are thus three-fold. (1) We improve the space environment simulator of previous work by implementing several potential future launch scenarios as well as debris mitigation strategies for newly launched satellites. (2) We provide a computationally efficient framework for exploring multi-actor policies for active debris removal. The core of the framework is a deterministic surrogate model of the orbital dynamics, which we have built using statistics collected from the improved, but still computationally expensive, full scale simulator. We validate this model and show it to be sufficiently accurate, thus allowing us to easily explore many different dynamic removal policies. (3) Using this framework, we compare the centralised solution with the decentralised one in terms of price of anarchy and evaluate the cost of several entities selfishly deciding on removal strategies. Furthermore, we investigate how the size of the space actors influences the strategy forming and its impact on social welfare.

This paper is structured as follows. Firstly, we position our study in the context of related work. Next, we present our space debris simulator which includes a collision model, a break-up model, an orbital propagator, and future launch scenarios. We then introduce and validate our surrogate model, vastly reducing the computational effort for predicting the impact of actions on the future space environment. Using this surrogate model we analyse the potential impact of several removal strategies on the orbital environment, and present a game theoretic analysis. Finally, we outline steps for further study, and conclude.

## 2. Related Work

This study significantly extends our previous work (Klima et al., 2016a), which was the first to study the space debris problem from strategic, game-theoretic point of view. Our extension is threefold; (i) we improve the space debris simulator by adding more realistic future launch scenarios and mitigation strategies, (ii) we develop a surrogate model allowing us to use dynamic strategies (compared to one-shot strategies in the previous work) and (iii) we use the price of anarchy to describe inefficiencies of various solution types.

Our work can be placed in the context of two different areas of related work. Firstly, from a simulation modelling perspective, various attempts have been made to accurately predict the evolution of space debris and the resulting risk of collisions for active spacecraft. Secondly, from a game-theoretic perspective, researchers have utilised similar methods to study related problems of environmental pollution, and the shared exploitation of scarce resources (Tahvonen, 1994).

One of the earliest analyses of the projected evolution of space debris was done by Donald J. Kessler in 1978 (Kessler and Cour-Palais, 1978; Kessler et al., 2010). This study led to the definition of the “Kessler Syndrome”, a particular scenario where the density of objects in LEO becomes high enough to cause a cascade of collisions, each producing new debris and eventually saturating the environment, rendering future space missions virtually impossible. In 2002, the Inter-Agency Space Debris Coordination Committee (IADC) outlined mitigation measures that should be implemented in newly launched spacecraft to limit the future growth of the debris population (Inter-Agency Space Debris Coordination Committee and others, 2002). While effective (Anselmo et al., 2001), it is now widely believed that mitigation alone is not enough to prevent a further build-up of the debris population in LEO (Liou and Johnson, 2008; Lewis et al., 2012, 2013).

As a result, *active debris removal* (ADR) methods, in which spacecraft are deployed to capture and de-orbit larger pieces of debris and out-of-service satellites, are now considered by many as a necessary step to ensure sustainability of LEO (Liou et al., 2010; Klinkrad, 2010). Several studies have been published recently in which the authors consider in detail the effect of active removal strategies to mitigate the space debris problem (Liou and Johnson, 2009; Liou et al., 2010, 2011). For example, Liou and Johnson (Liou and Johnson, 2009) present a sensitivity analysis on several fixed object removal strategies. They propose removing 5, 10, or 20 objects per year, and compare these mitigation strategies with baselines “business as usual” or “no new launches” and show the effectiveness of object removals. The objects to be removed are chosen according to their mass and collision probability. We base our study on Liou and Johnson’s approach but, in contrast, consider a more adaptive scenario in which an optimal strategy for removal can be *learned* based on estimated collision risks and removal costs. In our model we implement more sophisticated object removal criteria based not only on the potential risk of collision but also on the expected number of new debris that would result from such a collision.

The space debris removal dilemma is in many ways similar to other environmental clean-up efforts that have been studied using game-theoretic tools in the past. For example, Tahvonen models carbon dioxide abatement as a differential game, taking into account both abatement costs and environmental damage (Tahvonen, 1994). More complex models have been studied as well, including for example the ability to negotiate emission contracts (Harstad, 2012). Another related model is the Great Fish War of Levhari and Mirman (1980). Although not the same as environmental clean-up, this scenario deals with shared use of a scarce common resource, which potentially leads to the same dilemma in game theoretic terms, known as the *tragedy of the commons* (Hardin, 1968). While the problem of space debris can be seen as a tragedy of the commons in the sense, it’s potential solution by joint effort of different space actors can be modelled as a *public goods game*, in which players jointly need to reach a threshold contribution level in order to produce a public good (clean space, in our setting). A special case of the public goods game, in which contribution of a single player is sufficient, is given by the *volunteer’s dilemma* (Diekmann, 1985). Here, theory dictates that an increase in the number of players decreases the chance of any one player contributing due to the temptation of free-riding, known in psychology literature as the *diffusion of responsibility*. The space debris removal dilemma presented here is more complex than both game theoretic models, as we allow for different contribution levels as well as different stakes between the players.

Each of the aforementioned studies has focused solely on a (simplified) mathematical model of the underlying system. In contrast, we use a complex simulator to obtain an approximate model which can then be used to study the outcome of various fixed strategies, as well as learn new dynamic strategies that may outperform the fixed ones. In addition, while most previous work treats the dilemma as a one-shot (or repeated) game, we here propose a more realistic scenario in which different strategy choices can be made at different points in time, which we model within the framework of stochastic games. We investigate the application of reinforcement learning methods to obtain efficient strategies in such games.

Recently, there has been work using the learning approach in tragedy of the commons problems, analysing the dynamics of cooperative solutions (Leibo et al., 2017) or (Perolat et al., 2017). These works assumed partially observable domains with potentially unknown underlying model whereas in this work we assume fully observable surrogate model known to all the players. Another related work studying cooperation in public goods games uses a version of reinforcement learning called directional learning to (mis)learn and achieve more cooperative outcomes deviating from Nash Equilibria (Nax and Perc, 2015). This method is studied in an evolutionary setting based on one-shot interactions, whereas we study a more complex stochastic game in which dynamics depend on sequences of actions taken by the players.

We also mention the related (interdisciplinary) body of work focusing on the evolution of cooperation in populations of self-interested agents, often modelled using methods from evolutionary biology or statistical physics (Perc et al., 2017). While those approaches help to better understand why cooperation happens in (human) society on a macro scale, here we focus on the adaptive learning process on the micro-level of individual players. Although parallels can be drawn [see e.g., Bloembergen et al. (2015)], this type of analysis fall outside the scope of our current study.

In this work we study the inefficiency of decentralised solution in the active debris removal. The main tool for such analysis is the price of anarchy (PoA), first introduced by Koutsoupias and Papadimitriou (1999), however the study of inefficiency of Nash equilibria is older (Dubey, 1986). For general introduction to inefficiency in non-cooperative games we refer the reader to work of Roughgarden and Tardos (2007). PoA has been used in many domains, to name a few we state selfish traffic routing in a congested network (Roughgarden, 2005) or auctions (Roughgarden et al., 2017). In our work we focus on a more restricted scenario with PoA evaluation, similar works *measure* PoA (Knight et al., 2017) or analyse division fairness (Aleksandrov et al., 2015).

## 3. The Simulator

In this section, we describe the details of a full scale simulator we developed^2^ to predict the impact of certain actions, such as active removal of a space debris object, on the future space environment and in particular the assets of each actor. The definition of this simulator essentially defines the rules of the game we will analyze later on.

As up to date no active debris removal strategies have been attempted yet, there is only very limited existing data on their cost and effect. Any impact of such action can only be simulated. Furthermore, the space environment, similarly to the climate on Earth, only changes over relatively large time scales of many decades. To measure any effect of current actions, it is necessary to simulate at least one century into the future. This of course introduces large uncertainties to the outcome as it requires modelling of human behavior, i.e., future launch activity, over the next century. Instead of attempting to predict one model for future human space activity, we extended our existing simulator (Klima et al., 2016a) to allow the flexible definition of several possible future launch scenarios as described in the following.

The simulator is built on top of the Python scientific library PyKEP (Izzo, 2012). PyKEP provides basic tools for astrodynamics research, including utilities to interface with online databases such as the SATCAT^3^ and TLE (two-line element set)^4^ databases, which provide orbital information on all active (not decayed) objects in the low earth orbit (LEO) regime we are studying. These databases provide the input to our simulator. PyKEP also provides an implementation of the SGP4 satellite orbit propagator (via libsgp4^5^), which we use extensively in this work.

In previous work we extended PyKEP with a collision and break-up model (Klima et al., 2016a, b), which for completeness are discussed again in Sections 3.1 and 3.2. In addition, we now include more flexible future launch schedule allowing multiple scenarios based on different potential trends, as well as mitigation guidelines for active assets as provided by the Inter-Agency Space Debris Coordination Committee (IADC) (Inter-Agency Space Debris Coordination Committee and others, 2002).

The simulation is stepped at a fixed time step (e.g., 5 days). We use the SGP4 propagator in PyKEP to update the position of all orbital elements in our catalogue. At the end of each time step, the following procedures are executed:

1. Decay (see Section 3.1)
2. Collisions (see Section 3.2)
3. Launches (see Section 3.3)

We develop several scenarios that govern the projected future launch schedule, these are further detailed in Section 3.4.

### 3.1. Decay

If the propagator determines that an object’s orbit has decayed below a threshold (i.e., semi-major axis *a* ≤ 100 km) it is automatically considered to have re-entered the atmosphere. In addition, each newly launched object has a decay time assigned to it. This is the time after which the object is assumed to have decayed and it is removed from the simulation. This is to simulate modern satellites with active end-of-life mitigation techniques such as de-orbiting devices or graveyard orbit parking. While we do not simulate those de-orbiting actions explicitly, we do want to ensure that after the given lifetime objects do disappear from the catalogue.

### 3.2. Collision and Breakup Model

To evaluate the probability of collision between objects we follow the *Cube* approach (Liou et al., 2003). The Cube approach samples uniformly in time rather than space and is thus compatible with any orbital evolution simulation as it does not impose assumptions on the orbital geometry. This is particularly important in LEO, where orbital progression is significant in the considered time frame. We use the SGP4 (Vallado et al., 2006) orbital propagator to calculate the evolution of the ephemeridis (i.e., position and velocity) of an orbiting object given its TLE description. Ephemerides of all objects are calculated at regular time intervals. Space is then partitioned by a regular 3D-lattice and for any pair *i, j* of objects that fall into the same volume, the collision probability is evaluated as follows:

$$P_{i,j}=s_{i}s_{j}V_{rel}\sigma U,$$

where *s_i_* = *s_j_* are the spatial densities of object *i* an *j* in the cube, *σ*= *π*(*r_i_ +r_j_*)^2^ is the cross-sectional collision area, *V_rel_* is the collision (relative) velocity of the two objects, and *U* is the volume of the cube. For each pair, a pseudo-random number *x* is generated from a uniform distribution over the interval [0, 1]; if *P_i,j_* > *x*, a collision event is triggered.

We use the NASA standard breakup model (Johnson et al., 2001) to generate the population of fragments resulting from a collision event. The NASA/JSC breakup model is a widely accepted stochastic model of the fragmentation process of in-orbit collisions and explosions based on multiple ground-tests and radar observations of past events.

The model provides distributions for size, mass and ejection velocity of the fragment population parametrised by total mass and collision velocity of the parent objects. The number of fragments larger than a characteristic length-scale follows a power-law, the area-to-mass ratio follows a multivariate normal distribution, and the ejection velocity is sampled from a log-normal distribution. For details we refer to the original paper (Johnson et al., 2001) as well as the description of the model in (Klinkrad, 2010). For each sampled fragment, we create a new TLE entry using the fragment’s osculating elements, and add it to the population of objects being propagated. Although the breakup model also covers explosions as well as non-catastrophic collisions, we only consider catastrophic collisions (i.e., events leading to complete disintegration) in this work.

### 3.3. Future Launch Model

In order to simulate the future space environment, a crucial ingredient is the modelling of future launch activities into orbit. Previous work in the field, as well as our previous study (Klima et al., 2016a), employs a simple “business as usual” launch model that simply repeats the launch sequence of a past period (e.g., one decade). The only adjustment made to accommodate technological advances is a potential speed-up of the launch sequence by scaling it to a shorter period in the future. The problem with this modelling is that it does not allow for disruptive innovation in space technology and space economy.

Our launch model instead aims to provide finer control of the future scenarios. Clearly it is not possible to predict the future for the next century, and our launch model does not pretend to do that. Instead, our simulator is built to allow for a variety of possible future launch scenarios by adjusting various parameters. While this does not say anything about the probability of each scenario, it does allow to analyse their potential impact on the space environment if they were to happen.

The simulator is based on discrete time steps. To model future launches, the key metric we use is the mass launched into LEO per year. This mass is continuously injected into the orbital environment by spreading it over four different classes of spacecraft, the relative distribution of which changes with time to model technological progress made.

#### 3.3.1. Mass Per Year

We choose to model the total mass launched per year into LEO using a quadratic function

(1) $$M_{tot}(t)=M_{2000}\cdot (1+\alpha {\left(t-2000\right)}^{2})$$

with *t* in years AD and *M*_2000_ = 200,000 kg the total mass launched in the year 2000 used as a baseline.

This function is purely heuristic and is meant to combine two effects: the increased launch capabilities becoming available, which increases the total mass launched per year, and miniaturisation of satellite technology, which reduces the need to launch large mass into LEO. As neither effect can be modelled with any certainty we opted for a simple function that has only one parameter *α*.

**Figure 1:**
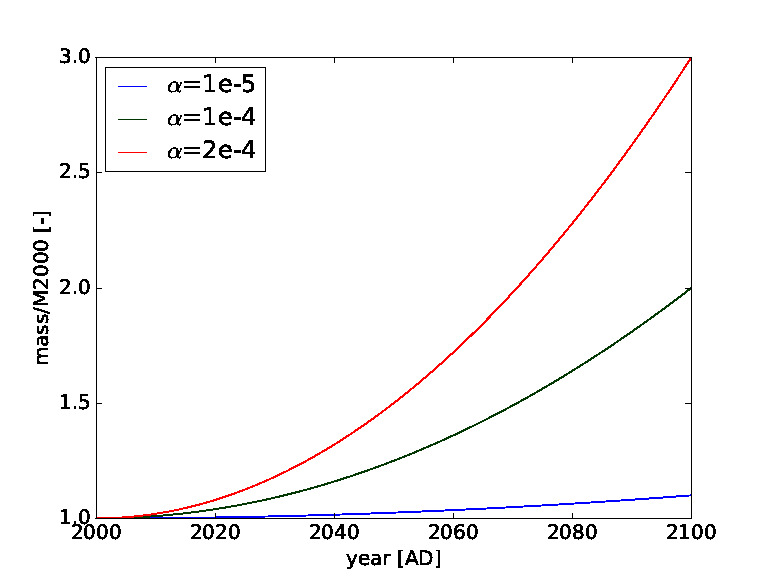
*Mass to orbit* defining the total mass launched per year into Low Earth Orbit in relation to baseline mass launched in year 2,000 (*M*2000) for different values of *α*. For example the green curve represents a doubling of the annual mass to orbit over the next 100 years.

The value of *α* allows to adjust the growth rate. Reasonable values would probably be around *α* = 10^–4^, leading to a doubling of the annual mass to orbit over the next 100 years, while *α* = 10^–3^ leads to a twenty-fold increase (see Figure 1). Negative values correspond to a decrease in launched mass over time, which could happen either due to technological advances making large launch mass unnecessary or a marked downturn in space activity.

#### 3.3.2. Spacecraft Classes

The following four classes of spacecraft are considered in our model:

- **Large satellites**, representative of the big communication and science satellites being actively launched and in common use today.
- **Medium satellites**, representative of newer science and technology demonstrators being developed and launched today and in the near future, e.g., for upcoming mega constellations.
- **Small satellites**, a group representative of cubesat type satellites which are being developed and tested today and may become increasingly attractive over the next decades.
- **Ultrasmall satellites**, a class of highly experimental nano-satellites, such as chipsats, envisioned to potentially become feasible in the future.

These classes are defined in terms of their attributes, including a typical mass and cost range for the satellite. More specifically, each class has the following attributes:

1. Time dependent market share,
2. Cost range,
3. Mass range,
4. Operational life time,
5. Decay time.

With the exception of the market share function, all other attributes are represented as a range of values sampled uniformly each time a new spacecraft is launched. These attributes then remain assigned to the newly instantiated spacecraft for the remainder of its lifetime. They represent the total cost of the spacecraft including launch, the total mass of the spacecraft (ignoring any differences between dry and wet mass), the date of the end of operational life and the date when this spacecraft is scheduled to decay and burn up in the atmosphere following a controlled deorbiting manoeuvre.

The market share is the share of the total number of newly launched satellites at a given time that belongs to this particular class. It is the only attribute that is an explicit function of time. The idea behind it is that right now there are still many large traditional satellites being launched, but that number will decrease as cubesat technology will mature and smaller satellites can perform the same functions as their larger predecessors.

To derive a market share function, we opted for a non-normalized Gaussian for each class:

$$g(t;\mu ,\sigma )=\exp-\frac{{\left(t-\mu \right)}^{2}}{2\sigma^{2}}.$$

where *t* is measured in years since the year 2000.

The two variables *μ*, indicating the center, and *α*, indicating the width of the distribution, are chosen for each class and form a crucial part of the scenario definition. They are heuristically chosen such that the final market share function exhibits the desired trend for a given scenario. Intuitively the centre *μ* can be thought of as the point in time at which the production of that class of satellites peaks, while the width *σ* determines the slope and length of the build-up and decline of that class.

At each moment in time *t*, the probability *p* of a newly launched satellite belonging to class *x*∈ *X*, i.e., the market share function for class *x*, is then given by the expression:

(2) $$p_{x}\left(t\right)=\frac{g_{x}\left(t\right)}{\sum_{i\in X}g_{i}\left(t\right)}$$

where *g_i_*(*t*) =*g*(*t; μ_i_, σ_i_*) is the Gaussian with the parameters for class *i*.

This definition of the market share function keeps the number of parameters defining the scenario sufficiently low, while providing enough flexibility to model different developments in the future. Examples for different scenarios and corresponding market share functions are given in Section 3.4.

#### 3.3.3. Orbits

The remaining attributes that need to be decided when launching new spacecrafts are the actual orbits to inject the spacecrafts into. SGP4 uses averaged Keplerian orbital elements (Vallado et al., 2006) for its orbit representation. In that representation, and restricting ourselves to the LEO regime, we arrive at the following bounds for newly launched satellites:

- **Semi-major axis:*** a *∈ [300, 1200] +*R_E_* km (distribution from current data)
- **Eccentricity:*** e *∈ [0.0, 0.5] (distribution from current data)
- **Inclination:*** i *∈ [0, 2* π*] (distribution from current data)
- **RAAN:** Ω ∈ [0, 2* π*] (uniform distribution)
- **Periapsis:*** ω* ∈ [0, 2* π*] (uniform distribution)
- **Mean anomaly:*** M *∈ [0, 2* π*] (uniform distribution)

The orbital elements semi-major axis (*a*), eccentricity (*e*), and inclination (*i*) are randomly chosen from the distribution of previously launched spacecraft. The rational for just replicating the current distribution is that those orbital parameters represent orbits that are chosen for astrodynamical reasons such as sun-synchronous orbits or polar orbits. As these features are based on the underlying physics, they will not change in the future and the same orbits can reasonably be expected to remain relevant depending on the objective of the satellite. The right ascension of the ascending node (Ω), argument of periapsis (*ω*) and mean anomaly (*M*), instead, just represent orbital orientation and position of the spacecraft within the orbit, and are less relevant for the astrodynamic properties of the orbit. They are therefore chosen from a flat distribution.

**Figure 2:**
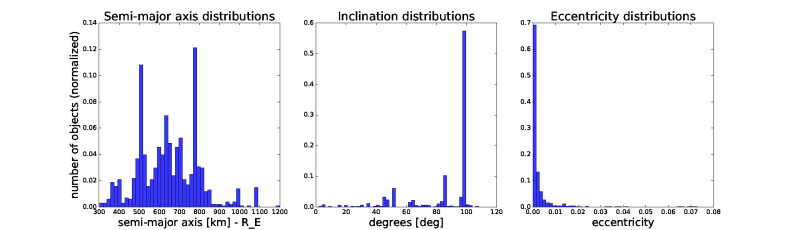
Distributions of semi-major axis, inclination, and eccentricity of objects filtered from current space catalogue from last 20 years. For launching we sample new objects’ orbital elements from these distributions. The remaining orbital elements are sampled uniformly at random from a given range.

In our simulation, we obtain the distributions for semi-major axis, eccentricity, and inclination from the current space catalogue filtered for objects in the past 20 years and within the given bounds, as shown in Figure 2.

The last input required for the SGP4 propagator is the so-called drag coefficient B* nominally defined as

$$B^{*}=\frac{\rho_{0}C_{D}A}{2m}$$

where *ρ*_0_ = 2.461 · 10^–5^ kg/m^3^ is the reference atmospheric density, *C_D_* is the drag coefficient, and *A/m* is the area-to-mass ratio. As these values are typically not known exactly, in practice B* is used to represent a range of non-conservative forces acting on the spacecraft. For real observations this parameter is typically fitted to provide the best agreement between SGP4 propagation and observation data. It is therefore possible to even find negative drag values in the satellite catalogue.

As B* is tightly related to the area to mass ratio *A/m*, and hence to spacecraft geometry, it is not possible to simply sample from previous distributions. As this parameter is used as a heuristic “catch-all” parameter in the SGP4 model, we simply set it to 0 for newly launched spacecraft. The justification for this is that for its active life a satellite will be maintained by its operator. This includes in particular orbit raising manoeuvres carried out regularly to maintain the operational orbit of a satellite. Similarly, as described above, our model assumes active end-of-life disposal of newly launched satellites by their respective operators. This eliminates the need for a drag term also during disposal.

For debris fragments generated during in-orbit collisions, on the other hand, B* does play a role in gradually decaying collision fragments. As the breakup model provides values for *A/m*, we simply assume a constant value of *C_D_* = 2.2, typically used for spacecraft where no other value has been determined experimentally, and compute B* from that.

#### 3.3.4. Launches

Instead of simulating individual launches, we regularly inject mass via averaged launches directly into the LEO environment. The justification for this is that while launches happen discretely, our simulation already ignores the rocket launcher itself, as well as initial commissioning and deployment phases of the satellites after being released by the launcher. Thus trying to predict individual launches does not add anything to the accuracy of the simulation.

A random set of new satellites is injected into orbit once per month, in our case the first time the simulation steps into a new month. The number of newly launched satellites is determined by the mass to orbit function evaluated at the current epoch. It provides the necessary information of how much mass to deliver to LEO each year, so dividing by 12 yields the newly launched mass to inject this month.

The spacecraft class of newly launched spacecraft is sampled from the probability distribution given by the market share functions following Eq. 2. The properties of each spacecraft are selected randomly within the parameter ranges defined for each class of spacecraft (Section 3.4). The orbits of the new spacecraft are chosen at random within the bounds specified by the global parameters of the simulation as detailed above. The spacecraft mass is then subtracted from the available launch mass. Note that even if the remaining launch mass is not sufficient, the spacecraft is still launched to avoid penalizing large spacecraft when dividing annual launch mass into monthly slices. As long as there is mass left, the process is repeated until all available launch mass has been used.

### 3.4. Future Launch Scenarios

To illustrate the launch model described above, we propose three scenarios that model the future nature of newly launched satellites. We chose these scenarios to illustrate three conceptually different developments in future launches with a time horizon of about 100 years. As mentioned before, we make no claim about how realistic these scenarios are, they are merely presented as possible developments in the future.

The three scenarios we propose are:

- **Conservative:** this scenario assumes little growth in space activity and is mostly “business as usual”. The total launched mass stays constant, and also technical progress is slow. Relatively few and large spacecraft are being launched during most of the century.
- **Moderate:** this scenario assumes moderate growth in space activity. Total mass launched increases moderately, doubling over the next 100 years. Some technical progress is being made, but the market share of large and mid-size satellites remains significant also at the end of the century.
- **Aggressive:** this scenario assumes aggressive growth both in space activity and technological development. Note that the mass to orbit in this scenario is actually decreasing slightly by about 10% as spacecraft miniaturization technology is developing fast enough to keep up with increased demand. By the end of the century, the vast majority of newly launched spacecraft are cubesats and clouds of futuristic chipsats.

**Figure 3:**
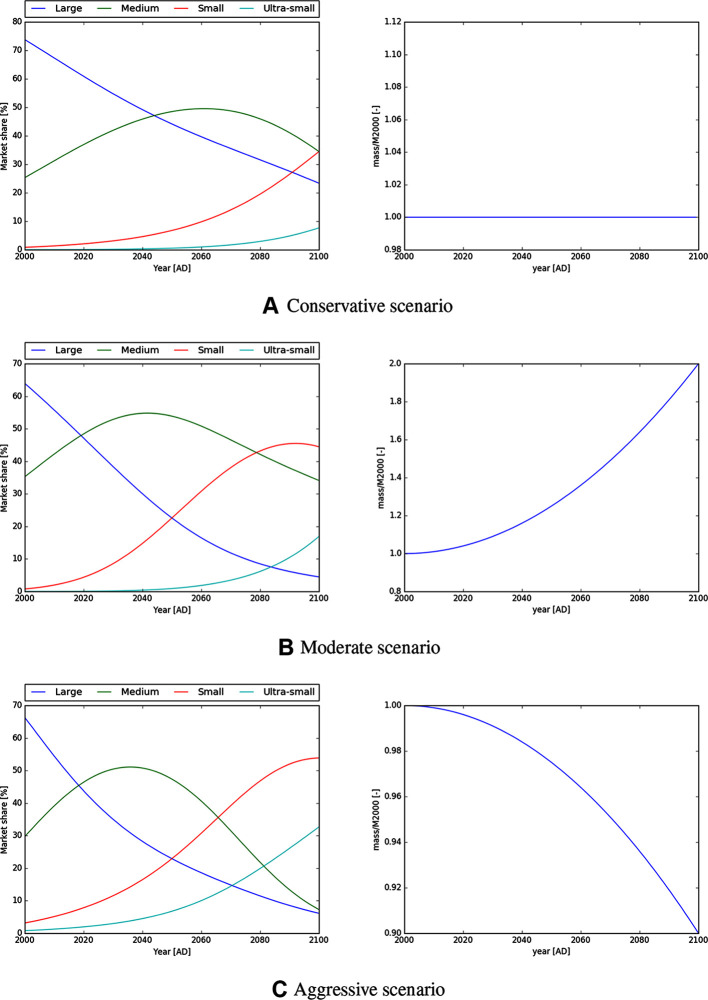
Market share and mass to orbit function for the conservative, moderate and aggressive launch scenarios as specified in Table 1. The conservative scenario assumes “business as usual” with constant launch mass and slower technical progress, however the aggressive scenario assumes a fast technological development with emphasis on miniaturisation of spacecrafts.

Table 1 lists the parameters corresponding to these scenarios. Figure 3 shows plots of the market share and total mass function for each scenario for illustration.

**Table 1:** Parameters for 4 different spacecraft classes (ultra-small, small, medium and large) and 3 launch scenarios (conservative, moderate and aggressive).

	**Ultra-small**	**Small**	**Medium**	**Large**
Cost range	[2k€, 1M€]	[1M€, 15M€]	[15M€, 40M€]	[40M€, 700M€]
Mass range	[0.1 kg, 10 kg]	[10 kg, 100 kg]	[100 kg, 500 kg]	[500 kg, 5,000 kg]
Operational time	[0.5 year, 1 year]	[0.5 year, 2 year]	[1 year, 5 year]	[10 year, 20 year]
Decay time	[0.5 year, 2 year]	[1 year, 7 year]	[7 year, 20 year]	[10 year, 25 year]
**Conservative**				
*μ*	2200	2150	2060	2020
*σ*	50	50	40	60
*α*	0			
**Moderate**				
*μ*	2150	2090	2060	1970
*σ*	35	30	50	60
*α*	10^–4^			
**Aggressive**				
*μ*	2150	2100	2040	1975
*σ*	50	40	30	60
*α*	–10^–5^			

Parameters μ, σ are defining the Gaussian launch function and parameter α the yearly mass launched to orbit.

## 4. Surrogate Model

The full scale simulator described above accurately models the space environment evolution given a launch model, but it is also computationally demanding. In order to facilitate efficient experimentation with different debris removal strategies we design an approximate surrogate model that effectively captures the dynamics of the system but is computationally fast. In this section we first describe the intuition and implementation of this surrogate model, after which we validate the approximation by comparing its projected dynamics with those given by the full scale simulator.

### 4.1. Implementation

**Figure 4:**
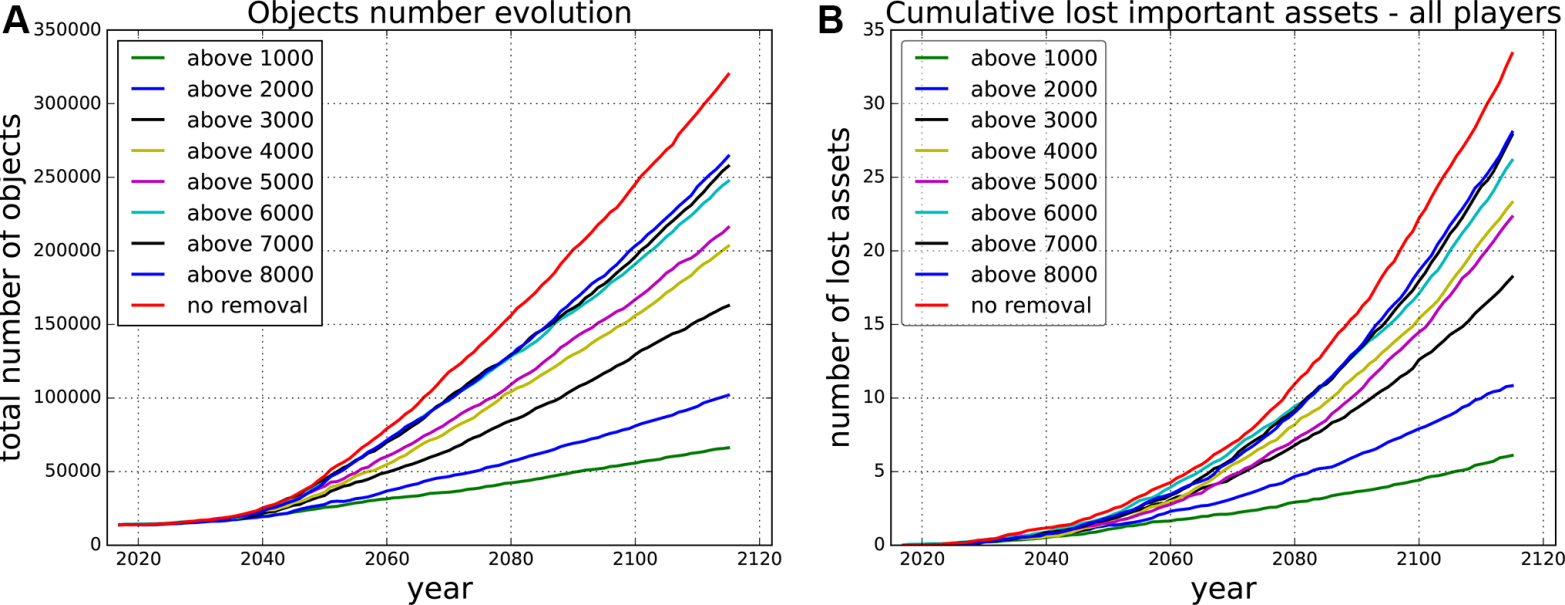
Projected evolution of the total number of objects **(A)** and cumulative lost assets **(B)** in the next 100 years for different removal strategies, e.g., *above 8,000* - removing all the objects causing in expectation a collision producing more than 8,000 debris pieces.

Firstly, we run Monte Carlo simulations for different settings of the *threshold for removal* of risky objects. We prevent all collisions that produce an expected number of debris larger than the given *threshold for removal* from happening by removing the risky objects causing the collisions. Figure 4 shows the evolution of the total number of objects in orbit for different thresholds for removal and the cumulative number of lost active assets for the same scenarios.

The outcome for every threshold setting in every time step can be evaluated by the gradient of the curve in that time step, which is based on the (expected) number of collisions (defining the number of objects injected into the environment, see Figure 4) and the (expected) number of lost active assets in every time step of the Monte Carlo simulations. We can use these two metrics to define the set of actions and the reward function.

We can view every point on every curve (system evolution for given threshold) as a potential system state. We restrict states to discrete time steps (decision points for policy change, e.g., 2 years) and assume that the system can transfer between the states by taking removal actions (joint action in the case of multiple actors) defined by given threshold for removal. Due to computational intractability we cannot run simulations for all combinations of (joint) actions and state transitions and hence we propose an approximate surrogate model for evaluating the effect of the removal action on the environment.

This model is based on transitions between the different curves depending on the actions taken. Note that the gradient of each curve is dependent on the total number of objects in the system and the year (which determines the number of active assets). Therefore, we propose *shifting* between curves either (i) horizontally – keeping the same level of total number of objects or (ii) vertically – keeping the same year and therefore the same number of active assets (remember that the number of active assets and their size distribution is only dependent on the launch scenario).

This *shifting* between the curves represents transitions between different states of the system. Note that the steeper the curve gets (larger gradient), the higher the risk for collisions will be. If players increase their effort, this means shifting to a lower curve, they move either down or right. A decrease in effort means moving up or left. Intuitively, increasing (decreasing) the effort should decrease (increase) the gradient.

Shifting down when increasing effort (or left when decreasing) gives us a lower bound on gradient of the curve, as we get an optimistic estimate of the further development of the system by underestimating the total number of objects (or the number of active assets when moving left) in the system. Following the same logic we get an upper bound on the gradient when moving right when increasing effort (or up when decreasing), which can be thought of as a pessimistic estimate where we overestimate the number of active assets (or the number of total objects).

We run 100 Monte Carlo simulations for each scenario to base our surrogate model on. The MC simulations give us for each time step and each threshold for removal the expected number of collisions (and their size in terms of the expected debris resulting from that collision). Basically, the players’ actions consist of removing (or deciding not to remove) the difference in expected number of collisions between two curves, and thus moving from the one to the other curve. As discussed, the way in which the system moves between curves can be defined to be either optimistic or pessimistic, to give a lower or upper bound on the expected number of collisions.

In the end, this results in a piece-wise combination of the different curves based on the removal actions taken. In Figure 4 never removing simply means sticking to the uppermost curve, always removing everything that would produce more than 1,000 debris pieces means sticking to the lowest curve, and any other combination leads to a mixture in between those two extremes.

### 4.2. Validation

**Figure 5:**
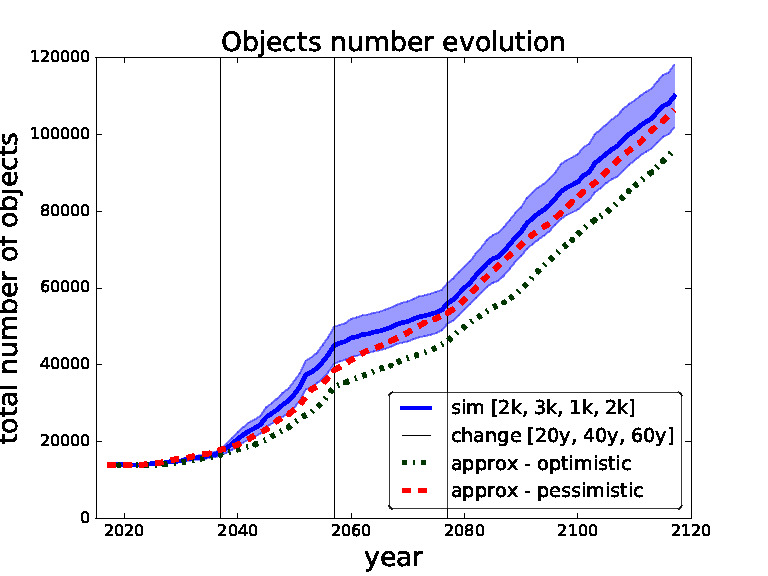
Validation for sequence of thresholds for removal - 2,000, 3,000, 1,000 and 2,000 changed after 20, 40 and 60 years. Comparing simulation with approximation.

**Figure 6:**
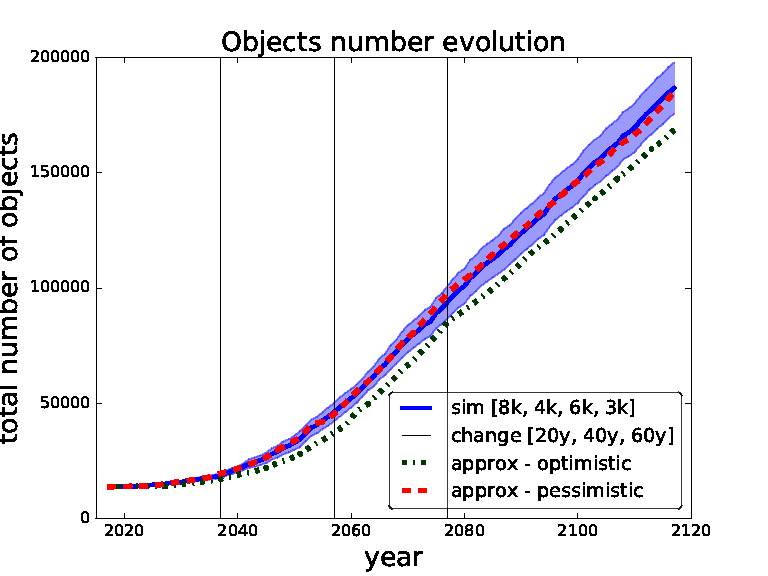
Validation for sequence of thresholds for removal - 8,000, 4,000, 6,000 and 3,000 changed after 20, 40 and 60 years. Comparing simulation with approximation.

**Figure 7:**
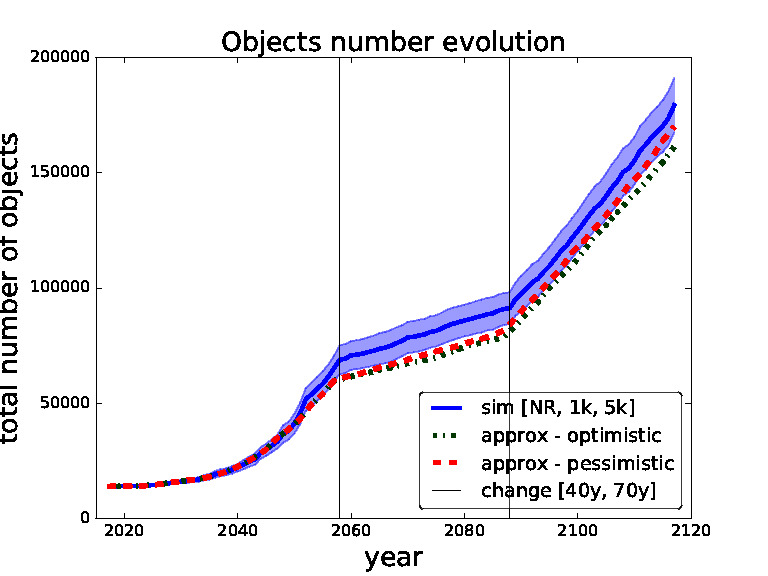
Validation for sequence of thresholds for removal - no-removal, 1,000 and 5,000 changed after 40 and 70 years. Comparing simulation with approximation.

**Figure 8:**
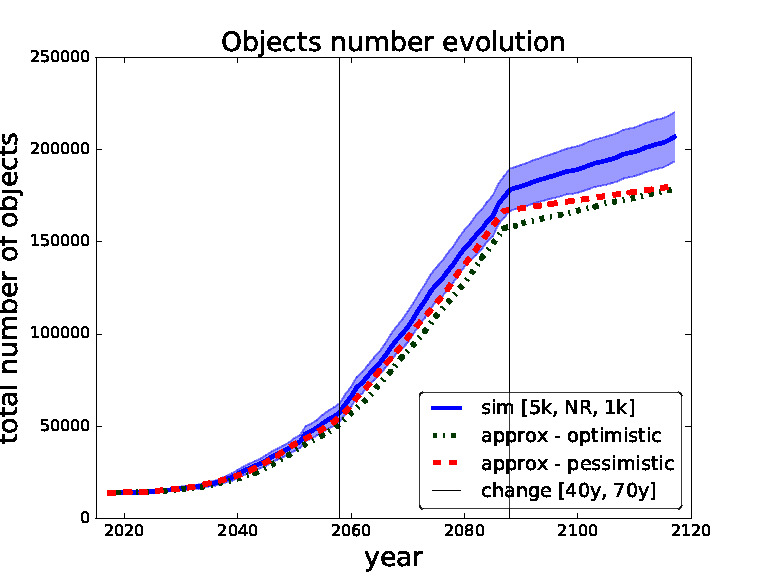
Validation for sequence of thresholds for removal - 5,000, no-removal and 1,000 changed after 40 and 70 years. Comparing simulation with approximation.

**Figure 9:**
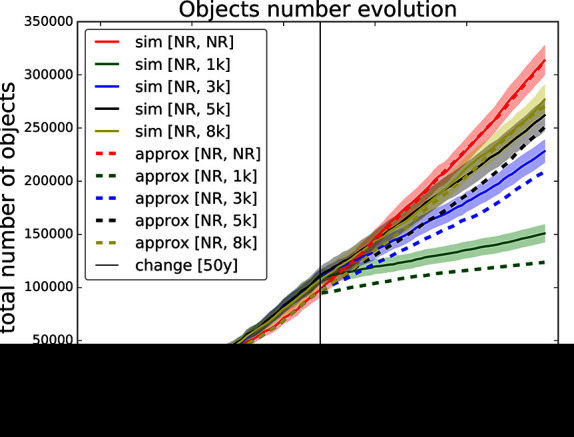
Validation for sequence of thresholds for removal - no-removal and [1,000, 3,000, 5,000, 8,000] changed after 50 years. Comparing simulation with approximation.

In order to validate our approximate surrogate model we compare outcomes of different settings of thresholds obtained from our surrogate model with the same settings obtained from the simulations of the full scale space debris model. In the following figures (Figures 5–8) the blue solid curves represent the simulations and the red dashed and green dash-dot curves represent the surrogate model (pessimistic and optimistic shifting respectively). In the Figure 9 the solid curves represent the simulations and the dashed curves represent the surrogate model. All the simulation curves have 95% CI plotted with respective colour shading. We investigate several combinations of *threshold for removals* with the focus on switching between the thresholds during the time horizon. Comparing switching between different thresholds demonstrates the robustness of our surrogate model. The black horizontal lines show the points of changing the strategy (threshold). Note that each simulation curve is run for 100 Monte Carlo runs and averaged over. We only show several settings to validate our surrogate model because of the high computational demands, where every Monte Carlo run takes on average 6 h^6^ i.e., each simulation curve taking approximately 600 h if run on a single thread.

We start with the setting of thresholds [2k, 3k, 1k, 2k] and changes [20*y*, 40*y*, 60*y*] in Figure 5. This setting represents a model where we use for the first 20 years threshold 2,000, then we change to threshold 3,000 for 20 years, then to threshold 1,000 for 20 years and then threshold 2,000 for the rest of the time horizon i.e., for another 40 years. We can see that the pessimistic approximation is most of the time within the simulation curve confidence bounds. In Figure 6 one can see the simulation and approximation for setting [8k, 4k, 6k, 3k] with the same intervals between switching. Again, the pessimistic approximation is within the confidence bounds of the simulation.

In Figures 7,8 we set the switching times to 40 years and then 70 years. We can see that in the first figure the pessimistic approximation is most of the time within the confidence bounds. In the second figure we can see that at the end even the pessimistic approximation leaves the CI, this is caused by abrupt switch from no-removal strategy to threshold 1,000 strategy (two extreme strategies in our model). We will later restrict our model to non-abrupt switching between the threshold strategies due to this behaviour.

Finally, in Figure 9 we compare several settings of thresholds with only one switch after 50 years. All the curves start with no-removal strategy and after 50 years they switch to one of the thresholds 1,000, 3,000, 5,000, 8,000 and no-removal i.e., continuing the no-removal strategy. One can observe that the abrupter the switch is the more the approximation curve deviates from the simulation curve. For example switching from no-removal to threshold 1,000 yields a significant deviation. On the other hand the approximation of switching from no-removal to threshold 8,000 stays within the confidence bounds of the respective simulation curve.

These experiments show that our approximate surrogate model – in the pessimistic setting – produces environment dynamics that fall within the 95% CI of the real Monte Carlo simulations most of the times (except for abrupt switching). This successfully validates our methodology, and means that we can use the surrogate model to efficiently compute environment evolution and resulting costs for various debris removal strategies. This will be discussed next.

## 5. Deterministic Game Model

We base the game model on our surrogate model as described above. We assume the model to be deterministic and think of it as a special case of a stochastic game with deterministic transitions.^7^ The game models situations in which multiple players interact. Each player selects an available action given the current state, and the game transitions to a next state based on the combination of actions taken. This makes stochastic games an intuitive framework within which to study strategic decision making between multiple parties, or multiple learning agents (Littman, 1994).

We start by modelling the environment as a deterministic Markov Decision Process (MDP), then move to a normal-form game formulation, and finally we define the combination of the two: a stochastic game model. The MDP model can be thought of as a single player approach. Building on that, assuming multiple players we arrive at the stochastic game model. Terminology typically differs between MDP, normal-form games, and stochastic games; in this paper we will use the terms player/agent, game/environment, and payoff/reward interchangeably.

### 5.1. MDP

In our case, the underlying environment dynamics are independent of the different players, and governed fully by the sum of actions taken (the chosen threshold for removal). As such, the stochastic game reduces to the special case of a Markov decision process (MDP). In order to transform the validated surrogate model into an MDP, we need to define the state space, the players, their actions space, the (immediate) reward function, and the state transition function. Formally, an MDP is defined by a tuple (*S, A, T, R, γ*), where *S* is state space, *A* action space, *T*(*s, a, s*ʹ) transition function assigning a probability to transiting from state *s *∈ *S* taking action *a *∈ *A* to a new state *s*ʹ ∈ *S, R*(*s, a*) a reward function for state *s* and action *a* and *γ* a reward discount factor. Each of these will be described in detail below.

The general intuition behind the MDP model is the following. At each time step (e.g., per year) players decide how much effort to invest next. This decision can be based on e.g., past actions, the current state of the environment, and budget limitations. The joint effort of all players determines the state evolution of the environment, i.e., the growth rate of the debris population for the coming time step (see model description above). An important question from a design perspective is whether effort will be treated as a discrete or continuous variable. The underlying model (as described above) is inherently discrete, based on a set of thresholds. The reward function can (naturally) be based on the expected number of lost assets between two time steps (states), plus the removal effort. If effort is expressed as an expected number of removals, this means we can get a monetary value by multiplying effort with the cost of removal.

#### 5.1.1. State Space

In principle the state space is infinite (continuous) along the dimensions of time and current number of objects in the environment (the two axis Figure 4). We discretise the state space along both dimensions: (1) by fixing the time steps to e.g., once every two years, and (2) by fixing the number of allowable states at each time step, e.g., uniformly between the top and bottom curves in Figure 4. This will fix the total number of states of the MDP.

#### 5.1.2. Action Space

As our surrogate model is based on a notion of threshold, i.e., by deciding not *how many* object to remove exactly but based only on the *impact* of the potential collision, it seems natural to base the action space on these thresholds as well. Thus, the action space is defined by the discrete thresholds, where for each such a threshold in any given time step we have the expected number of removed objects *E*(*n_rem_*) and the expected number of lost assets *E*(*n_lost_*) during the next time step. This data come from the Monte Carlo simulations.

The definition of the action space partially defines how the cost associated with each action will be defined. In this case, the cost in terms of future losses is directly given by the effort (threshold) curves in Figure 4; however the cost of removals will vary (“everything above a threshold” can be any number of removals, and this will vary over time).

#### 5.1.3. Reward Function

The (joint) reward function is naturally given by the cost of lost assets plus the cost of removal efforts. Minimizing these costs means maximizing reward.^8^ The reward of the underlying MDP can be also thought of as the environment welfare, which we use in our experimental analysis.

We define the reward function *R*(*s, a*) for state *s* and action *a* as a sum of the cost of losing assets and the cost of object removal efforts in the next time interval:

(3) $$R\left(s,a\right)=E\left(n_{lost}\right)+\lambda \cdot E\left(n_{rem}\right)$$

where $\lambda =\frac{C_{R}}{C_{L}}$ is a ratio between cost of removal *C_R_* and cost of losing an asset *C_L_*. In this paper we do the analysis for different levels of *λ* as we are only interested in the relation between cost of removal and cost of losing an assets and not in their actual values, which can be difficult to determine. *E*(*n_lost_*) is the expected number of lost assets in the next time interval and *E*(*n_rem_*) is the expected number of removed objects in the next time interval defined by the action *a* in environment state *s* (given by the threshold curves in the surrogate model).

Of importance is the fact that the system has an infinite horizon, whereas our simulation results use a finite horizon. Therefore it might be necessary to derive a (heuristic) evaluation of the final simulation state that captures somehow the expected future rewards. For example, we can assume that the last immediate reward (or an average over the past *x* rewards) provides an estimate for the future, and hence take as evaluation of the final state an infinite discounted sum over that (average) reward.

#### 5.1.4. Transition Function

The transition function of our MDP is defined by the underlying surrogate model. Given the action *a* and current environment state *s*, the transition function *T*(*s, a*) deterministically returns the successor state *s*ʹ. The function follows the *curve shifting* method explained above.

### 5.2. Normal-Form Game

One of the key models in game theory is the normal-form game, which is also known as the matrix or strategic form (Leyton-Brown and Shoham, 2008). A finite n-person game is defined as a tuple (*n, A, R*), where *n* is a finite number of players, *A*_1_, …, *A_n_* is a finite set of actions for each player and *R*_1_, …, *R_n_* is a reward function for each player. The standard way to represent such a game is by an n-dimensional matrix, where for every combination of players’ actions a reward for each player is given. A crucial concept in game theory is the Nash equilibrium (Nash, 1951), which we also use in our analysis. In a Nash equilibrium every player chooses the *best response* to the actions of the other players involved in the game. In other words Nash equilibrium is a situation where no player can do better by unilaterally changing his strategy.

### 5.3. Stochastic Game

Stochastic game (SG) (Shapley, 1953) generalises Markov decision processes (MDP) and repeated games. SG is a game model with multiple agents moving in environment defined by states, actions, rewards and (stochastic or deterministic) transition function, defined as a tuple (*n, S, A*_1_, …, *A_n_*, *R*_1_, …, *R_n_, T*) where *n* is number of agents in the system, *S* is a finite set of system states, *A_k_* is the action set of agent *k*, *R_k_ : S* × *A*_1_ × … × *A_n_* × *S* → R is the reward function of agent *k* and *T : S* × *A*_1_ × … × *A_n_* × *S* → [0, 1] is the transition function. We will define the components of Stochastic game based on the underlying MDP as described above.

#### 5.3.1. Players

Players are defined solely by their size, expressed in terms of their number (share) of active assets. This number in turn determines their risk given the current state of the environment. Since we do not discriminate between objects of different players in our model, we assume that the risk scales linearly with the number of assets owned by the players. Each player *i* has a share *ξ*(*i*) of assets representing the size of the player. The value of *ξ*(*i*) is in range (0, 1) and represents the proportion of player assets to all assets in the environment, thus ∑*_ n_ ξ*(*i*) = 1.

#### 5.3.2. State Space

State space is identical to that of the MDP model. Note that due to multiple players the Markov property is broken. Nevertheless we still apply learning methods to derive players strategies. We assume the players can fully observe the underlying state i.e., the time period and the total number of objects in the system.

#### 5.3.3. Action Space

Defining the joint effect of several individual actions in the multi-player game is not straightforward. We define a joint action **a** as a sum of removal efforts of the players, which is a sum of the expected number of removals for each player’s chosen threshold. Then we map this sum to a joint threshold. For this joint threshold we obtain a total expected number of removed objects and total expected number of lost assets from the underlying MDP. We then proportionally divide the expected number of removed objects to each player according to their expressed effort. Such method will enable to define individual rewards.

#### 5.3.4. Reward Function

We define the reward function based on the MDP reward function definition in Section 5.1. The reward function *R*(*s*, **a**) for state *s* and joint action **a** is defined as a sum of the cost of losing assets and the cost of object removal efforts in the next time interval, which is equal to sum of all players’ rewards:

(4) $$\begin{array}{ll} R\left(s,a\right) & =E\left(n_{lost}\right)+\lambda \cdot E\left(n_{rem}\right)\\ & =\sum_{i}R\left(s,a_{i}\right)=\sum_{i}\left(E\left(n_{lost}^{i}\right)+\lambda \cdot \left(n_{rem}^{i}\right)\right) \end{array}$$

*E(n^i^_lost_)* is the expected number of lost assets in the next time interval for player *i* and *E*(*n^i^_rem_*) is the expected number of removed objects in the next time interval for player *i*.

Individual reward for player *i* is defined as $R\left(s,a_{i}\right)=\xi \left(i\right)\cdot E\left(n_{lost}\right)+\lambda \cdot E\left(n_{rem}^{i}\right),$ where *ξ* (*i*) is the share of important assets of player *i*.

#### 5.3.5 Transition Function

The transition function is defined according to the underlying deterministic MDP based on the surrogate model. Given the joint action $\vec{a}$ and current environment state *s*, the transition function $T(s,\vec{a})$ deterministically returns the successor state *s*ʹ. The function follows the *curve shifting* method explained above.

## 6. Decision Making

We look at two types of decision making differing in complexity. Firstly, we focus on the one-shot scenario with *static* strategies, similar to the method used in (Klima et al., 2016a), where the players fix their strategies at the beginning of the simulation and stick with those until the end. Secondly, we analyse *dynamic* strategies, where the players can dynamically decide on their action in every time step based on other players’ past actions and the development of the environment.

Furthermore we analyse and compare a single-agent and multi-agent model. Thus, we can divide the analysis into four types of decision making: (i) single-agent static, (ii) multi-agent static, (iii) single-agent dynamic and (iv) multi-agent dynamic. We discuss these variants in turns.

### 6.1. Single Agent

Firstly, we assume only one agent (player) in the system. An entity of n cooperating players can be thought of as a single agent, where every action of the agent is a joint action of the players defined as $\vec{a}=f(a_{1},\ldots ,a_{n})$, where *f* is a function aggregating several actions into one joint action. Having only one agent in the system we can directly solve the underlying MDP to find an optimal strategy for the agent. The optimal strategy is given in the form of a sequence of actions across the time horizon *T* as π* = (*a*_1_, …, *a_T_*). Since the state transition function *T* is assumed to be deterministic in our surrogate model, applying strategy π* to the MDP will give us a fixed sequence of states and fixed sum of (discounted) rewards.

We differentiate two levels of complexity for the optimal strategy. Firstly, we consider a static strategy, where one fixed action is repeated for the complete duration until the time horizon, as in previous work (Klima et al., 2016a). Secondly, we consider a dynamic strategy, which can consist of different actions taken at different time points. In this case the agent can dynamically change action during the course of the MDP until reaching the final (goal) state.

The optimal strategy for the static case can be found by simply maximising the sum of rewards over the strategy space, which has the size of the discrete action space |*A*|. Moving from the static to the dynamic case, the problem of finding the optimal strategy becomes more complicated. Now, the strategy space consists of all possible sequences over all discrete actions, which is of size |*A*|*^T^* for time horizon *T*. To find the optimal strategy we have to solve the underlying MDP, which can be done by dynamic programming for a small strategy space, or by reinforcement learning for a large strategy space.

### 6.2. Multiple Agents

From a single agent scenario we move to a multi-agent scenario. We consider *n* agents (players), and analyse the interaction among the players over the underlying MDP by using the game model. We assume the players do not cooperate and are self-interested – in the case of cooperation we can model the problem as a single agent scenario as described above. Again we are interested in finding optimal strategies for the players; however optimality in a multi-agent scenario can be defined in various ways. One way to define optimality is by finding equilibria solutions, another way is by maximising the global welfare. In this paper we consider and compare both approaches.

As in the previous section we differentiate between two levels of complexity in the decision making process. Firstly, we look at static strategies, defined as sequences of a repeated fixed actions. In the multi-agent scenario this can be described by a normal-form game and solved by finding Nash equilibria of this game. We are interested mainly in pure equilibria strategies, because mixed strategies are typically difficult to maintain in real-world settings.

Secondly, we analyse the case of dynamic strategies, where the players can take different actions in every time step. The solution is a sequence of actions for each of the players. The strategy space for *n* players is large even for a small action space and short time horizon, and grows exponentially with them. As a result, solving the resulting stochastic game explicitly quickly becomes intractable. Thus, the only feasible way how to find optimal or near-optimal solutions is to approximate them using e.g., reinforcement learning.

#### 6.2.1. Learning an Optimal Strategy

In the space debris removal decision making process we face the problem of *delayed reward*, where the effect of immediate actions (object removal or passivity) will only fully come into effect only after many years, making reward-based decision making difficult. Temporal difference methods solve the delayed reward problem by bootstrapping, i.e., building iteratively more accurate models by incorporating expected future returns into the learnt reward function (Sutton and Barto, 1998). Typically a state value function *V*(*s*) or state-action value function *Q*(*s, a*) is learned which describes the expected optimal future return given a current state *s* (and action *a*).

For example, the Q-learning algorithm (Watkins and Dayan, 1992) iteratively updates the function *Q*(*s, a*) as:

(5) $$Q\left(s,a\right)=\left(1-\alpha \right)Q\left(s,a\right)+\alpha \left(R\left(s,a\right)+\gamma \cdot \underset{a^{\prime }}{max}\;Q\left(s^{\prime },a^{\prime }\right)\right)$$

where *s*ʹ and *a*ʹ are the next state and action, respectively, and *γ* ∈ [0, 1] discounts future rewards. In this case, the state *s* might include more than just the system state, depending on whether the player has knowledge of other players’ actions. If so, a history of recent play might augment the individual player’s state space representation, thereby making it exponentially larger.

### 6.3. Evaluation Metrics

In this paper we want to analyse and compare different decision making models. The decision making is based on the underlying MDP built on the surrogate model. Therefore, the main evaluation metrics are based on the concept of reward. The decision making process is prescribed by a given strategy, which can be evaluated in terms of (discounted) rewards both from an individual perspective as well as from a global (environment) perspective. In the analysis we use the concept of social welfare *ω*, which is described as sum of all players’ rewards and can be thought of as the environment outcome.

We use the social welfare to compare the above stated approaches using the concept of *Price of Anarchy* (PoA) (Koutsoupias and Papadimitriou, 1999), which measures efficiency between two sets of solutions, *S*_1_ and *S*_2_, where the latter is assumed to be worse than the former and is defined as:

(6) $$PoA=\frac{\max_{s\in S_{1}}\omega (s)}{\min_{s\in S_{2}}\omega (s)}$$

In our experiments the welfare *ω* is always negative, thus we redefine the PoA to get *PoA* ≥ 1 as:

(7) $$PoA=\frac{\min_{s\in S_{2}}\omega (s)}{\max_{s\in S_{1}}\omega (s)}$$

For strategies from strategy spaces *S*_1_ and *S*_2_, *ω* is the environmental welfare or also the MDP reward for joint strategy of the players. We use this metric to compare outcomes from different model designs. In our experimental analysis we use two variants of Price of Anarchy to measure efficiency, (i) PoA between single-agent and multi-agent as a price for selfish behaviour of the agents and (ii) PoA between static and dynamic strategy as a price for not being able to flexibly react to changes in the environment. We notate the first as *PoA_m_* (single-agent vs. **m**ulti-agent) and the second as *PoA_d_* (static vs **d**ynamic).

We define the concept of *fairness* in space debris removal game based on player *i* share *ξ*(*i*) as described in Section 5.1. *Fairness* is based on the assumption that a level of the removal effort should be proportional to the size of the player. We define *fairness* as $\phi \left(i\right)=\frac{\omega \cdot \xi \left(i\right)}{r\left(i\right)}$, where *r*(*i*) is a reward for player *i*. If *ϕ*(*i*) = 1 we say that player *i* behaves fairly, if *ϕ*(*i*) > 1 we say that player *i* behaves positively unfair, meaning he has higher reward than he deserves (removing less than what would be fair for his share of assets) and if *ϕ*(*i*) < 1 we say he behaves negatively unfair, meaning he has lower reward than he deserves (removing more than what would be fair for his share of assets). We also define total fairness as a sum of differences from fair case for each player *i* as *ϕ* = ∑*_ i_* |1 – *ϕ*(*i*)|. The total fairness describes the quality of a solution. If *ϕ* = 0 we have a fair solution, the greater the value of *ϕ* is the less fair solution we have. In Table 2 we state a list of notations to help the reader to better orientate in the following sections.

**Table 2:** List of notations used in the game model.

*PoA_m_*	Price of anarchy comparing single-agent and multi-agent scenario
*PoA_d_*	Price of anarchy comparing static and dynamic scenario
*λ*	Ratio between cost of removal *C_R_* and cost of losing an asset *C_L_*
*ξ*(*i*)	Share of important assets of player *i*, i.e., size of player *i*
*ω*	Welfare, i.e., sum of all players’ rewards
*ϕ*(*i*)	Fairness for player *i*
*ϕ*	Total fairness

In Section 7 we experimentally compare different scenarios using these evaluation metrics. We perform a thorough analysis for different levels of the ratio *λ* and shares of assets *ξ*(*i*).

## 7. Experiments and Results

We base all our experiments on the surrogate model, which is build on the data from Monte Carlo runs of the space debris simulation model. Thus, we have the expected number of lost assets *E*(*n_lost_*) and expected number of removed objects *E*(*n_rem_*) for every time step and every *threshold for removal* (see Figure 4). The *threshold for removal* is defining the discrete action space over a set {1,000, 2,000, 3,000, 4,000, 5,000, 6,000, 8,000, 9,000, 10,000, *NR*}, where *NR* is “no debris removal” as defined in Section 5.1. The previously described surrogate model can be used to efficiently compare various (predefined) debris removal strategies, investigate the effect of various parameter choices, and to learn an optimal strategy automatically. Our surrogate model is based on *curve shifting* as described in Section 4, in order to approximate well we restrict the players from abruptly changing the removal thresholds, meaning they can only shift one up, one down or stay at the same threshold level in every time step. The initial choice of action (threshold) is arbitrary. In this section we describe several experiments that highlight each of these possibilities in turn. In all our experiments we assume 100 years time horizon and decision time step 10 years, so we have 10 decision making points. The time horizon is based on the ISO standard on space debris mitigation (International Organization for Standardization, 2011) agreed to by all space agencies. It sets the time frame for graveyard orbit stability simulations to 100 years. Given the generally quite long lead time for space missions, averaging about 7.5 years for governmental satellite operations (Davis and Filip, 2015), the 10 year decision time step seems a reasonable time for space actors to decide on strategy.

As described in previous section we analyse several scenarios. Our goal is to compare solution quality of static vs. dynamic scenario and single-agent vs. multi-agent scenario and combinations of these. We first need to describe how we can obtain these scenarios. We will see that some can be computed and some need to be learned due to complexity. We firstly demonstrate and describe the different scenarios on fixed settings of (i) *λ* = 0.1, which is the ratio between cost of removal *C_R_* and cost of losing an asset *C_L_* and (ii) in the multi-agent case the share of important assets *ξ*(*A*) = 0.6 of player *A*. Then, we perform a thorough analysis of the scenarios for different settings of parameters *λ* and *ξ* and describe the influence of these parameters on a quality of the solution.

Following is the list of the scenarios and corresponding methods how to find an optimal strategy:

- Single-agent, static → iterate over solutions and find the one with maximal reward
- Single-agent, dynamic → solve MDP directly by dynamic programming
- Multi-agent, static → find optimum by computing Nash equilibrium
- Multi-agent, dynamic → learn optimum by using reinforcement learning e.g., temporal difference algorithm

### 7.1. Static Strategies

We start with a static strategy, which is one action fixed for the entire time horizon i.e., 100 years. A static strategy can be written down in form of dynamic strategy using the MDP, where at every time step the player chooses the same action. The final reward will be obviously the same.

#### 7.1.1. Single-Agent

Firstly, we look on a system with a single agent. Getting optimal strategy for a single-agent static scenario is straightforward due to discrete and small action space. We have |*A*| possible strategies for which we compute the rewards and choose the one with the highest reward. In Table 3 we show the optimal strategies for different levels of *λ*. They are optimal in sense of maximising the total reward (payoff) defined as *R* = –(*n_lostAssets_ + λ* · *n_removed_*).

**Table 3:** Optimal single-agent static strategies for different parameter *λ*, where the strategy is fixed for the entire time horizon.

**λ**	0.1	0.2	0.3	0.4	0.5
Strategy	3000	5000	5000	9000	9000
Total reward	−23.3867	−27.9443	−30.0443	−31.9917	−32.74

For increasing λ (i.e., object removal gets more costly) the optimal strategy is to remove fewer objects (i.e., greater threshold for removal).

#### 7.1.2. Multi-Agent

We now move to a multi-agent scenario with static strategies (see Section 6). This scenario can be written down as a normal-form game, the pure strategies are defined by the *threshold for removals*. For a normal-form game the solution concept is Nash equilibrium.

In our analysis we assume two-players A and B. The players differ only in size defined by share of important assets as described in Section 5.1. We assume that agent *i* has a share *ξ*(*i*) of total important assets from all important assets in the environment. The reward (payoff) for player A is defined as $R_{A}=\xi \left(A\right)\cdot E\left(n_{lost}\right)+\lambda \cdot E\left(n_{rem}^{A}\right)$ and for player B as $R_{B}=\left(1-\xi \left(A\right)\right)\cdot E\left(n_{lost}\right)+\lambda \cdot E\left(n_{rem}^{B}\right)$. Note that *ξ*(*A*) = 1 – *ξ*(*B*). The pure strategies are the *thresholds for removal* which give us the values of *E*(*n_lost_*) and *E*(*n_rem_*). We are now able to construct the payoff matrix for given constants of *λ* and *ξ*(*A*).

We demonstrate forming the payoff matrix for *λ* = 0.1 and *ξ*(*A*) = 0.6. We describe the entries in the payoff matrix for player A choosing pure strategy “*threshold for removal* = 2,000” as expected number of removed objects $E(n_{rem}^{A})$ and player B choosing pure strategy “*threshold for removal* = 6,000” as $E(n_{rem}^{B})$. We obtain the values of $E(n_{rem}^{2000})$ and $E(n_{rem}^{6000})$, which come from the simulation data. We compute the value of *E*(*n_lost_*) by finding a threshold curve for given joint action which is a sum of removal efforts as described in Section 5.3. Then, we compute the payoff matrix entries *R_A_* and *R_B_* for the chosen pure strategies as $R_{A}=0.6\cdot E\left(n_{lost}\right)+0.1\cdot E\left(n_{rem}^{A}\right)$ and $R_{B}=0.4\cdot E\left(n_{lost}\right)+0.1\cdot E\left(n_{rem}^{B}\right)$. In Table 4 we can see the corresponding Nash equilibria, the strategy is written in format [<*player* >: <*action* >, <*probability* >]. We show all the pure equilibria (1, 2, 3) and one mixed equilibrium (4), note there exists more mixed equilibria, however we show only one. In general we are interested only in pure strategies, because in space debris removal problem it is unfeasible to play mixed strategies. For each Nash equilibrium we show player A reward *R_A_*, player B reward *R_B_*, the welfare *ω* (the sum of rewards) and the fairness *ϕ*.

**Table 4:** Optimal multi-agent static strategies, where the solution concept is Nash equilibria.

*NE*	1	2	3	4
Strat	**A**: 4k,1 **B**: NR,1	**A**: NR,1 **B**: 5k,1	**A**: 6k,1 **B**: 10k,1	**A**: 4k,0.36; NR,0.64 **B**: 4k,0.61; 9k,0.39
*R_A_*	−16.19	−14.25	−15.80	−14.13
*R_B_*	−8.64	−11.60	−10.09	−10.89
*ω*	−24.83	−25.84	−25.90	−25.02
*ϕ*(*A*)	0.920	1.088	0.984	1.062
*ϕ*(*B*)	1.150	0.891	1.027	0.919
*ϕ*	0.230	0.197	0.043	0.143

We show player A’s and B’s rewards, welfare and fairness for parameter λ = 0.1 and share parameter ξ(A) = 0.6 (i.e., player A owns 60% of all assets). There are three pure Nash equilibria and several mixed ones (we show only one mixed NE in the last column).

### 7.2. Dynamic Strategies

We can now move to dynamic strategies as described in Section 6. The players can decide on action every time step. Thus, the strategy is defined as a sequence of actions over the time steps. Allowing the agents to dynamically shape their strategy is more realistic than fixing the strategy through the course of the time horizon. However, dynamic strategies are severely more complex, making the whole interaction with the system and potentially with other players much more complicated. As stated before we assume time horizon 100 years with decision time steps 10 years, thus having 10 decision points, where the agent(s) have to choose an action. We describe and experiment with the single-agent and multi-agent case in turns.

#### 7.2.1. Single-Agent

In the single-agent case the optimal strategy is obtained by solving the underlying MDP. This is a strong property of the proposed model; we can optimally plan the strategy given the surrogate model. For small state spaces we can iterate over the whole space and find optimal strategy, for larger state spaces we can use dynamic programming and for even larger state spaces we can use reinforcement learning methods. We show in Table 5 the optimal strategies for different levels of parameter *λ*. The strategies are shown as a sequence of actions, e.g., the optimal strategy for *λ* = 0.1 is choosing the action “*threshold for removal* 3,000” in every time step. We compare Table 5 with Table 3 and can see that the dynamic strategies are better than (or at least as good as) the static strategies in terms of total reward, this results is intuitive because the player has more flexibility in the dynamic case.

**Table 5:** Optimal single-agent dynamic strategies for different parameter *λ*.

***λ***	Strategy	Welfare
0.1	3k,3k,3k,3k,3k,3k,3k,3k,3k,3k	−23.39
0.2	5k,5k,5k,5k,4k,4k,4k,4k,5k,4k	−27.49
0.3	9k,8k,6k,5k,5k,5k,6k,5k,5k,5k	−29.69
0.4	9k,9k,10k,NR,10k,9k,9k,9k,10k,9k	−31.47
0.5	10k,NR,NR,NR,10k,10k,9k,9k,10k,9k	−32.05

For increasing λ (i.e., object removal gets more costly) the optimal strategy is to remove fewer objects (i.e., greater thresholds for removal) and the welfare increases.

In multi-agent scenario or in the case of larger state space it might be unfeasible or computationally demanding to explicitly solve the MDP and compute the optimal strategy. Thus, we focus on learning the optimal strategy. We use a standard reinforcement learning method Q-learning as described in Section 6.2.1. In Table 6 we show the single-agent dynamic strategies which we learned using Q-learning and compare them with the optimal ones from Table 5 to validate the learning method. In bold we can see the differences to the optimal strategies and in the last column we state the difference in total reward between the learned and the optimal strategies. We can see that especially at the beginning the learned strategies might differ, this is caused by rather similar threshold curves behaviour at the beginning of the time horizon (see Figure 4). We can conclude that the learning method is successful and we will use it for further analysis in the multi-agent scenario.

**Table 6:** Learned single-agent dynamic strategies for different *λ*..

***λ***	Strategy	*R*	Δ
0.1	3k,3k,3k,3k,3k,3k,3k,3k,3k,3k	−23.39	0%
0.2	**4k**,**4k**,5k,5k,4k,4k,4k,4k,5k,4k	−**27.64**	0.5%
0.3	**6k**,**8k**,6k,5k,5k,5k,6k,5k,5k,5k	−29.69	0%
0.4	**10k,NR,NR**,NR,10k,**10k**,9k,9k,10k,9k	−**31.52**	0.2%
0.5	10k,NR,NR,NR,10k,10k,9k,9k,10k,9k	−32.05	0%

Differences to the optimal strategies from Table 5 are shown in bold and differences in rewards are stated in the last column. We can successfully validate the learning process due to high similarity to the optimal strategies.

#### 7.2.2. Multi-Agent

In the space debris removal problem there can be several space actors interacting with each other and deciding on removal strategy. Therefore, from a single-agent we arrive to a multi-agent dynamic scenario. We now face very difficult problem of finding the optimal strategies due to *moving target problem* and potentially very large state space. Thus, we focus on learning the optimal strategies. We firstly show learning a strategy against a fixed opponent (opponent playing a static strategy) and then learning a strategy against a learning strategy. In our analysis we assume two players, which we denote player A (primarily the learning agent) and player B (opponent). However the players differ only by their share of important assets *ξ*, which represents the space actor size. We make here a model design assumption of identical space program of different space actors differing only in size, i.e., homogeneous spacecraft types, spacecraft sizes, used orbits etc.

#### Against Fixed Opponent

We assume the opponent (player B) to have a fixed strategy, which is one of the possible thresholds; this is a static strategy as described above. We show in Table 7 learned strategies against different fixed strategies. In the first column we state the opponent (player B) fixed strategy, e.g., 3 k means the player B will choose in every time step the threshold 3,000. We compare two types of learned strategies for the player A: (i) altruistic strategy, which maximize the environment welfare and (ii) selfish strategy, which maximizes the player A reward. In the table we also state the players’ rewards *R*(*i*), welfare *ω*, price of anarchy *PoA* between altruistic and selfish behaviour of player A and fairness *ϕ*. We can see that the price of anarchy is similar for most of the fixed strategies. This means that once the opponent fixes his strategy the environment welfare can be improved by approximately 5–6% whether we play selfishly or altruistically. Finally we show the fairness *ϕ* as described in Section 6. We can observe that the selfish behaviour is fairer compared to the altruistic, which is expected. We can also see that some of the fixed strategies give very bad environment welfare e.g., “fixed 1 k” gives more than double loss.

**Table 7:** Optimal multi-agent dynamic strategies against fixed opponent for parameter *λ* = 0.1 and share parameter *ξ*(*A*) = 0.6.

Fixed B	Type	Strategy	*R*(*A*)	*R*(*B*)	*ω*	PoA	**ϕ**
1k	altr	10k,NR,NR,NR,NR,NR,NR,NR,NR,NR	−3.70	−43.27	−46.97	-	7.19
1k	self	10k,NR,NR,NR,NR,NR,NR,NR,NR,NR	−3.70	−43.27	−46.97	1	7.19
3k	altr	10k,NR,NR,NR,NR,NR,NR,NR,NR,NR	−10.54	−12.85	−23.39	-	0.60
3k	self	10k,NR,NR,NR,NR,NR,NR,NR,NR,NR	−10.54	- 12.85	−23.39	1	0.60
5k	altr	3k,3k,4k,4k,3k,4k,3k,3k,3k,3k	−14.64	−8.75	−23.39	-	0.111
5k	self	10k,NR,10k,9k,8k,8k,8k,6k,6k,6k	−14.07	−10.58	−24.65	1.054	0.115
6k	altr	3k,3k,3k,3k,3k,3k,3k,3k,3k,3k	−15.14	−8.25	−23.39	-	0.207
6k	self	5k,5k,5k,6k,5k,6k,6k,5k,6k,6k	−14.65	−10.11	−24.76	1.059	0.034
8k	altr	3k,3k,3k,3k,3k,3k,3k,3k,3k,3k	−15.52	−7.87	−23.39	-	0.285
8k	self	5k,5k,6k,6k,5k,5k,5k,4k,5k,4k	−15.07	−9.61	−24.68	1.055	0.045
10k	altr	3k,3k,3k,3k,3k,3k,3k,3k,3k,3k	−15.83	−7.56	−23.39	-	0.351
10k	self	5k,5k,6k,5k,4k,4k,4k,4k,5k,4k	−15.44	−9.24	−24.68	1.055	0.109
NR	altr	3k,3k,3k,3k,3k,3k,3k,3k,3k,3k	−16.36	−7.02	−23.39	-	0.475
NR	self	5k,5k,5k,5k,4k,4k,4k,4k,5k,4k	−15.93	−8.75	−24.68	1.055	0.199

We show optimal altruistic (altr) and selfish (self) strategies. In the first column we show the opponent (player B) fixed strategy. We state the rewards, welfare, fairness and price of anarchy between different solutions. We can see that fixed strategies can lead to very sub-optimal solutions.

#### Learning Against Learning Strategy

We discussed before that the multi-agent scenario is too complex to compute the optimal strategy. Therefore, we now investigate the dynamics of two players learning each other’s strategy. We assume both players learning the strategy by using the standard Q-learning. We assume discount factor *λ* = 1 i.e., no discount, the learning rate *α* = 0.01, the exploration parameter *ε* = 0.1. We discretize the state space as described in Section 5.1 to debris levels with step size 1,000 and the time step of 10 years. We learned all the strategies over 1 million episodes. Both players can observe the state and the Q-values are independent on the other player action, thus this learning can be seen as independent Q-learning, which is a common method in multi-agent reinforcement learning (Bloembergen et al., 2015).

In Table 8 we show several learned strategies for a single setting of the parameters *λ* = 0.1 and *ξ*(*A*) = 0.6. We can see the strategies for player A and B, their rewards *R*, the welfare *ω* and the individual and overall fairness *ϕ*. One can note that these strategies have lower welfare *ω* than the worst pure Nash equilibrium welfare in static scenario (Table 4). We can compare these strategies in terms of fairness *ϕ* or welfare *ω*, where these two metrics are not necessarily dependent on each other. A better welfare does not mean fairer division of removal efforts.

**Table 8:** Learned multi-agent dynamic strategies using Q-learning against Q-learning opponent with parameter *λ* = 0.1 and share parameter *ξ*(*A*) = 0.6.

Player	Strategy	*R*(*i*)	**ω**	**ϕ**(*i*)	**ϕ**
A	6k,6k,5k,6k,5k,4k,4k,4k,5k,4k	−15.51	−24.75	0.957	0.113
B	9k,8k,6k,8k,8k,9k,10k,NR,NR,NR	−9.25		1.070	
A	6k,5k,4k,4k,4k,4k,4k,4k,4k,4k	−16.00	−24.76	0.929	0.202
B	9k,10k,9k,10k,NR,NR,NR,NR,NR,NR	−8.76		1.131	
A	6k,8k,6k,5k,4k,4k,4k,4k,5k,4k	−15.84	−24.83	0.941	0.164
B	10k,9k,9k,10k,NR,NR,NR,NR,NR,10k	−8.99		1.105	
A	6k,8k,8k,6k,5k,4k,4k,4k,5k,4k	−15.62	−24.91	0.957	0.116
B	9k,10k,9k,9k,8k,9k,10k,NR,NR,NR	−9.29		1.073	

We show four different outcomes of the same setting. We can attain highly effective solutions using Q-learning for both players.

So far we have shown the learning for fixed size of the players represented by parameter share *ξ*. We now investigate how different levels of *ξ* influence the solution and its quality. We show such an analysis in Table 9. We experiment with 9 different divisions of shares of important assets between the two players, in the first column we show the shares *ξ* for each of the players. We can see that the less a player owns the less he wants to remove and vice versa which is expected. One can see that for the cases when a player owns only a small proportion of the assets he prefers not to remove anything e.g., *ξ*(*i*) = 0.1. Very important outcome from this table is the evolution of the environment welfare, one can observe that for disproportional players we get higher welfare than for proportional players (compare welfare of *ξ*(*A*) = 0.1 to welfare of *ξ*(*A*) = *ξ*(*B*) = 0.5, –23.39 and −25.07 respectively). Another important outcome is how the size of the players influence the fairness. Looking at the Table 9 we can note that the more similarly sized the players are the fairer strategy they can learn. In the case of very disproportional players, they learn very unfair strategy e.g., *ξ*(*i*) = 0.1 or *ξ*(*i*) = 0.2. For equally sized players, the learned strategy is the most fair.

**Table 9:** Learned multi-agent dynamic strategies using Q-learning against Q-learning opponent with parameter *λ* = 0.1 and different levels of assets share *ξ*(*i*).

**ξ**(*i*)	Player	Strategy	*R*(*i*)	**ω**	**ϕ**(*i*)	**ϕ**
0.1	A	10k,10k,NR,NR,NR,NR,NR,NR,NR,NR	−1.76	−23.39	1.329	0.356
0.9	B	3k,3k,3k,3k,3k,3k,3k,3k,3k,3k	−21.63		0.973	
0.2	A	10k,10k,NR,NR,NR,NR,NR,NR,NR,NR	−3.57	−23.41	1.311	0.368
0.8	B	3k,3k,3k,4k,3k,3k,3k,3k,3k,3k	−19.85		0.943	
0.3	A	10k,NR,NR,NR,NR,NR,NR,NR,NR,NR	−6.42	−24.47	1.143	0.194
0.7	B	5k,5k,4k,5k,4k,4k,4k,4k,4k,4k	−18.05		0.949	
0.4	A	NR,NR,10k,NR,NR,NR,NR,NR,NR,NR	−8.78	−24.68	1.124	0.193
0.6	B	5k,6k,5k,5k,4k,4k,4k,4k,5k,4k	−15.90		0.931	
0.5	A	6k,8k,8k,6k,6k,5k,6k,6k,8k,6k	−12.46	−25.07	1.006	0.012
0.5	B	8k,8k,8k,6k,5k,6k,6k,5k,6k,5k	−12.61		0.994	
0.6	A	6k,8k,8k,6k,5k,4k,4k,4k,5k,4k	−15.62	−24.91	0.957	0.116
0.4	B	9k,10k,9k,9k,8k,9k,10k,NR,NR,NR	−9.29		1.073	
0.7	A	5k,4k,4k,5k,4k,4k,4k,4k,4k,4k	−18.12	−24.55	0.948	0.197
0.3	B	NR,10k,NR,NR,NR,NR,NR,NR,NR,NR	−6.43		1.145	
0.8	A	3k,2k,3k,3k,3k,3k,3k,3k,3k,3k	−20.08	−23.58	0.939	0.405
0.2	B	NR,10k,NR,NR,NR,NR,NR,NR,NR,NR	−3.51		1.344	
0.9	A	3k,3k,3k,3k,3k,3k,3k,3k,3k,3k	−21.63	−23.39	0.973	0.356
0.1	B	NR,NR,NR,NR,NR,NR,NR,NR,NR,NR	−1.76		1.329	

Starting from highly disproportional players in the top row to equally sized players in the middle row. High disproportion in the players’ size attains high welfare but trades off for fairness, where the small sized player removes barely anything.

### 7.3 Analysis and Comparison

We have presented several scenarios using the surrogate model and have described methods how to find efficient strategies for them. We now compare the different solutions of those scenarios in terms of quality and efficiency. In Table 10 we show the different scenarios and their values of welfare *ω* and fairness *ϕ*. We state single-agent, multi-agent, static and dynamic scenarios and the methods to find respective effective strategies. For every scenario we state a code, which we use in further analysis. Note that the optimal strategies were obtained by exhaustive search (brute force) and the learned strategies by Q-learning. All the shown combinations of scenarios were discussed in turn in the previous sections. We compare here only the scenarios for parameters setting of *λ* = 0.1, *ξ*(*A*) = 0.6 and time step 10 years in 100 years horizon. For some of the scenarios we obtained several solutions, thus we state maximal and minimal values of welfare and fairness. We can see that playing against a fixed opponent can cause a high unfairness, which is expected due to the non-optimal fixation of removal effort.

**Table 10:** Comparison of different scenarios in terms of welfare *ω* and fairness **ϕ** for **λ** = 0.1 and share parameter **ξ**(*A*) = 0.6.

Code	Agents	Type	Obtained	**ω*_max_*	**ω*_min_*	**ϕ*_ min_*	**ϕ*_ max_*
SO1	1	static	optimal	−23.39	−23.39	-	-
DO1	1	dynamic	optimal	−23.39	−23.39	-	-
DL1	1	dynamic	learned	−23.39	−23.39	-	-
SO2	2	static	optimal (NE)	−24.83	−25.90	0.043	0.230
FO2	2	dyn/fixed	optimal	−23.39	−46.97	0.034	7.19
DL2	2	dynamic	learned	−24.75	−24.91	0.113	0.202

We show combinations of single-agent, multi-agent, static and dynamic approaches which were obtained either by learning or by exact computation. In case there were multiple solutions for given scenario we present maximal and minimal values.

We want to now compare these different scenarios; how efficient they are. For such comparison we use the concept of price of anarchy *PoA* as described in Section 6. We assume two types of *PoA*, the first type *PoA_m_* compares the single-agent scenario with the multi-agent one, i.e., the cost for having self interested (competing) players instead of centralised (single-agent) strategy and *PoA_d_* which compares the static scenario with the dynamic one. *PoA_d_* can be thought of as the advantage we get by playing dynamically i.e., being able to change the strategy in every time step. In Table 11 we compare all the scenarios (code names from Table 10) in terms of price of anarchy for fixed *λ* = 0.1 and *ξ* (*A*) = 0.6. Of interest are the values in bold and in italic, which show price of anarchy *PoA_d_* between the static and dynamic scenario and price of anarchy *PoA_m_* between the single- and multi-agent scenario, respectively. One can note that the cost of using a static strategy over a dynamic one (SO2 vs. DL2) is 4.7% for the multi-agent case and 0% for the single-agent case (SO1 vs. DO1). The cost of multi-agent scenario to single-agent scenario is 5.8% for the dynamic case (DO1 vs. DO2) and 10.7% for the static case (SO1 vs. SO2), which is caused by the selfish behaviour of the players. Also note the high values of *PoA* of the multi-agent fixed scenarios (FO2), meaning that a fixed strategy can cause a highly inefficient outcome in the terms of the environmental welfare.

**Table 11:** Comparison of different scenarios in terms of price of anarchy *PoA* for *λ* = 0.1 and share parameter *ξ*(*A*) = 0.6.

Code	SO1	DO1	DL1	SO2	FO2	DL2
SO1	1	**1**	1	*1.107*	2.008	1.058
DO1	**1**	1	1	1.107	2.008	*1.058*
DL1	1	1	1	1.107	2.008	1.058
SO2	*1.107*	1.107	1.107	1	1.814	**1.047**
FO2	2.008	2.008	2.008	1.814	1	1.898
DL2	1.058	*1.058*	1.058	**1.047**	1.898	1

The codes of the scenarios are stated in Table 10. In **bold** we show *PoAd* (static vs. dynamic) and in *italic* we show *PoAm* (single-agent vs. multi-agent). Note that for example *PoA* = 1.107 means 10.7% inefficiency.

We have shown the methodology of comparison of different scenarios for fixed parameters of *λ* and *ξ*. In the next section we investigate the quality of the scenarios for varying levels of those parameters.

#### 7.3.1. Varying Levels of *ξ* and **λ**

We investigate the different scenarios and corresponding optimal solutions for different settings of the two main parameters studied (i) ratio *λ* between the cost of removal *C_R_* and the cost of losing an important asset *C_L_* and (ii) share of important assets *ξ*. We do the analysis for *λ* ∈ [0.1, 0.2, 0.3, 0.4, 0.5] and *ξ* ∈ [0.1, 0.2, 0.3, 0.4, 0.5]. As stated before the players differ only in the size expressed by the parameter *ξ*, thus the results for *ξ*(*A*) = 0.4 and *ξ*(*B*) = 0.6 in two player case are interchangeable with *ξ*(*A*) = 0.6 and *ξ*(*B*) = 0.4. Obviously, this holds for any setting of *ξ*.

We run the experiments for the 4 main scenarios; single-agent static, single-agent dynamic, multi-agent static and multi-agent dynamic. The methods to obtain optimal strategies for these scenarios are discussed in the previous sections. The comparison metric is the price of anarchy *PoA*. We distinguish between comparing single-agent with multi-agent scenarios using refined *PoA_m_* and static with dynamic scenario using *PoA_d_*.

Firstly, we compare single-agent scenarios, in Table 12 we show the welfare *ω* and *PoA_d_* for static and dynamic scenarios for different levels of *λ*. We can observe that by using the dynamic strategy we can improve the environment welfare by up to 2.2% (in the case of *λ* = 0.5).

**Table 12:** Comparing static and dynamic single-agent scenarios in terms of welfare **ω** and price of anarchy *PoA_d_* for varying parameter *λ*.

**λ**	0.1	0.2	0.3	0.4	0.5
static	−23.39	−27.94	−30.04	−31.99	−32.74
dynamic	−23.39	−27.49	−29.69	−31.47	−32.05
*PoA_d_*	1	1.016	1.012	1.017	1.022

Note that for increasing *λ* (i.e., cost of removal becomes more expensive) the welfare decreases and the difference between a static anda dynamic scenario increases.

Moving to the multi-agent case in Table 13 we firstly show the optimal solutions to static scenario obtained by computing the Nash equilibria. We mentioned before that we are only interested in the pure Nash equilibria, thus in the case of multiple pure equilibria for given parameters *ξ*(*A*) and *λ* we show only the minimal and maximal values of those in the Table 13. One can see that some of the values are repetitive, this is caused by limited flexibility of the static solutions and potentially by the available actions. For instance, the high values of parameter *λ* mean that it is very expensive to remove objects compare to losing assets, meaning that the players prefer to remove as few as possible e.g., no-removal strategy hence some of the constant welfares in the table. One can see that the values of welfare *ω* decrease with increasing values of *λ* and with more equally sized players expressed by the share parameter *ξ*. Although, this conclusion is achieved only experimentally, it does strongly suggest such trend. The similarly sized players cause inefficiency of the environment welfare due to being selfish. As discussed before obtaining the optimal strategies for the single-agent scenario is not computationally as demanding as for the multi-agent scenario, where we might not be able to compute the optimal strategy but need to learn it. In Table 13 we state the resulting welfares *ω* for multi-agent dynamic scenario and varying levels of the studied parameters. We can again see the same trend; with increasing *λ* and *ξ* the welfare worsens.

**Table 13:** Comparison of static and dynamic multi-agent scenarios in terms of welfare *ω* for different levels of λ and share parameter *ξ,* The static scenario is obtained by computing Nash equilibria and the dynamic scenario is learned using Q-learning.

	ξ(*A*)\λ	0.1	0.2	0.3	0.4	0.5
Nash Eq. static	0.1	−23.39	−27.94	−30.04	−31.99	−32.74
	0.2	−23.39	−27.94	−30.04	−31.99	−33.56
	0.3	-23.39/–25.90	−27.94	−31.24	−31.99	−33.56
	0.4	-24.83/–25.90	−27.94	−31.24	−33.56	−33.56
	0.5	-25.84/–25.90	-28.05/–30.50	−31.24	−33.56	−33.56
Q-learned dynamic	0.1	−23.39	−27.65	−29.72	−31.53	−32.07
	0.2	−23.41	−27.71	−29.74	−31.54	−32.06
	0.3	−24.91	−27.86	−30.99	−31.53	−32.86
	0.4	−24.77	−27.86	−30.99	−31.65	−32.34
	0.5	−25.47	−28.22	−31.17	−32.02	−32.86

In case of multiple solutions we state maximal and minimal values (multiple NE). Note that with increasing parameter λ (object removal becomes more expensive) and increasing ξ (the players become more equally sized) the welfare decreases. One can see the improvement in welfare of dynamic strategies compared to the static ones.

We now have the welfare *ω* values for all the scenarios and all the settings of the studied parameters. We are interested in comparing them in terms of price of anarchy *PoA*, which expresses the inefficiency between different scenarios. We start with *PoA_m_* comparing single-agent static (Table 12) with multi-agent static (Table 13) scenarios in Table 14. We can see that the inefficiency induced by having multiple players is ranging from 0% to 10.7%. One can observe that the inefficiency grows with more equally sized players, which is to be expected.

**Table 14:** Comparison of single-agent and multi-agent static scenarios in terms of price of anarchy *PoA_m_* for varying levels of share parameter **ξ** and parameter **λ**.

***ξ**(*A*)\*λ**	0.1	0.2	0.3	0.4	0.5
0.1	1.000	1.000	1.000	1.000	1.000
0.2	1.000	1.000	1.000	1.000	1.025
0.3	1.107	1.000	1.040	1.000	1.025
0.4	1.107	1.000	1.040	1.049	1.025
0.5	1.107	1.092	1.040	1.049	1.025

We can observe the increasing inefficiency of solutions for increasing ξ (the players become more equally sized).

From static scenarios we move to comparing dynamic scenarios, in Table 14 we show the analysis of *PoA_m_*, comparing single-agent dynamic with multi-agent dynamic scenario. We obtain inefficiencies ranging from 0% to 8.9%. One can again see that *PoA_m_* grows with more equally sized players, where we get the highest values for the same sized players i.e., *ξ*(*i*) = 0.5. This is the cost pay for competing selfish agents compared to having centralised solution i.e., a single entity deciding on removal effort.

Finally, we look at *PoA_d_* between multi-agent static (NE) and multi-agent dynamic (Q-learned) in Table 15. As expected the dynamic solutions are better then the static ones, except for the setting *λ* = 0.1 and *ξ*(*A*) = 0.2, which is caused by learning only sub-optimal strategy. We can expect that with increased number of episodes 881 we would obtain better dynamic strategy than in the static case even for this setting of the parameters. We can observe that the solutions of static vs. dynamic scenarios differ from 0% to 8.1%. Thus, the inefficiency in the multi-agent scenario induced by being limited to a static strategy compared to a dynamic strategy can be up to 8.1%.

**Table 15:** Comparison of single-agent dynamic vs. multi-agent dynamic and multi-agent static vs. multi-agent dynamic scenarios in terms of price of anarchy (*PoA_m_* and *PoA_d_*) for varying levels of share parameter *ξ* and parameter *λ*.

	***ξ***(*A*)\λ	0.1	0.2	0.3	0.4	0.5
*PoA_m_*	Single-agent dynamic vs. multi-agent dynamic					
	0.1	1.000	1.006	1.001	1.002	1.001
	0.2	1.001	1.008	1.002	1.002	1.000
	0.3	1.065	1.013	1.044	1.002	1.025
	0.4	1.059	1.013	1.044	1.006	1.009
	0.5	1.089	1.027	1.050	1.018	1.025
*PoA_d_*	Multi-agent static vs. multi-agent dynamic					
	0.1	1.000	1.010	1.011	1.015	1.021
	0.2	0.999	1.008	1.010	1.014	1.047
	0.3	1.040	1.003	1.008	1.015	1.021
	0.4	1.046	1.003	1.008	1.060	1.038
	0.5	1.017	1.081	1.002	1.048	1.021

Note that in the comparison of the single-agent dynamic vs. multi-agent dynamic scenarios for increasing parameter ξ (more equally sized players) the inefficiency increases.

## 8. Conclusion

We have made several contributions in this paper to the state of the art in the field. The main contributions are three-fold. (1) A significant improvement of the space debris environment simulator from the previous work (Klima et al., 2016a), (2) Developing an efficient surrogate model of the computationally expensive simulator enabling the study of multi-actor policies for active debris removal. We validated this model and showed it to be sufficiently accurate, thus allowing us to easily explore many different dynamic removal policies. (3) Extensive comparison of the centralised solution with the decentralised one in terms of price of anarchy and evaluation of the cost of several entities selfishly deciding on removal strategies. We summarise these contributions in turn in the following.

This paper greatly extends a previous version of our space debris simulator (Klima et al., 2016a) built on the PyKEP scientific library, In addition to two existing datasets on currently known space objects, a flexible launch model predicting future launch activity has been integrated based on feedback received from the European Space Agency. This replaces the previous “business as usual” model of repeating previous launch activity into the future by a more flexible model of future launch activity. We furthermore significantly extended the game theoretical analysis of previous work (Klima et al., 2016a), which pioneered the strategic, game-theoretic approach to space debris removal problem. In particular, where the previous work (Klima et al., 2016a) only considered a static one-shot interaction in the form of a normal-form game, we investigated dynamic strategies and multiple agents, and employed reinforcement learning techniques to study the resulting high dimensional complex strategic interaction. This is a novel contribution in the field of debris removal, where previous studies on the cost of removal consider either the effect of cooperatively removing individual objects or using simple, fixed strategies for each actor (Liou and Johnson, 2009; Liou et al., 2010, 2013).

Using statistics from extensive Monte Carlo roll-outs using the developed full simulator we proposed a computationally efficient surrogate model that accurately captures the dynamics of the space debris environment for various debris removal strategies. Unlike other surrogate models in the literature (Lewis et al., 2009), we derive our surrogate model by curve-fitting the full simulation results including various launch models and accurately simulated orbital motion. This ensures that our surrogate model faithfully represents our full simulation, without the potential bias introduced by a specific choice of surrogate model parameters, such as a fixed insertion rate of debris.

We have shown various ways in which this surrogate model can be used to study the effect of different strategies. In addition, we have formulated a stochastic game based on the surrogate model, which we used to study multi-party decision making. As an example, we have shown how machine learning techniques (here, Q-learning) can be used to learn an optimal debris removal strategy that outperforms fixed strategies.

We have compared and evaluated both a single-agent and a multi-agent approach to the problem of space debris removal. By computing the Price of Anarchy we analysed the cost of decentralised (individually rational) decision making as compared to a centrally optimised strategy. Our results showed that such cost can be up to 10.7% in the static case and up to 8.9% in the dynamic case depending on the parameters of ratio *λ* between cost of removal and cost of losing an important asset and share *ξ* of important assets defining the size of the players. We can see that the cost of decentralised solution is quite significant, considering the enormous level of resources needed for the space debris removal. Thus, the space actors should aim to minimize the number of competing agents in the environment by for example forming coalitions.

Furthermore, we investigated the difference between static strategies and dynamic strategies. Static strategies have the advantage of simplicity of the decision making, but are less effective than their dynamic counterparts. We compared both in terms of price of anarchy. In the single-agent case, the cost of using a static strategy is up to 2.2%, and for the multi-agent case the cost is up to 8.1% depending on the setting of parameter λ.

Comparing single-agent vs. multi-agent scenarios and static vs. dynamic scenarios we showed that the parameter *ξ* – the share of important assets, representing the size of the players – has a big impact on quality of the solution. The more similarly sized the players are the less efficient solutions we obtain, i.e., equally sized players produce the worst solutions. On the other hand, highly disproportional players arrive to more efficient solutions and the values of price of anarchy *PoA* for single-agent vs. multi-agent and static vs. dynamic scenarios are equal or very close to 1, meaning there is no or low inefficiency.

We were also interested in fairness of the players’ strategies depending on their size. The idea of fairness was driven by the assumption that the level of the removal effort should be proportional to the size of the player. In our analysis we defined the concept of fairness and described how the size of the players (given by their number of assets) influences the final outcome in terms of global welfare and fairness. We found out that the more equally sized the players are the fairer strategy can be learned at the cost of reduced global welfare. On the other hand, the more disproportional the players are the better global welfare they can attain, at the cost of a more unfair distribution of effort. This realisation is in line with the increasing price of anarchy for more selfishly acting players.

This result in particular might serve to inform policy and decision making processes. A coordinated, global approach towards space debris removal, effectively reducing to one single actor, may be more effective in maximizing the effect on the space environment than the current, distributed approach of various actors acting independently. Such a global entity for space debris removal could be set up through international agreements with proportional contributions by different actors, thus maintaining fairness while achieving a maximum of impact.

Finally, we propose several directions for future work that can be facilitated by the simulator and surrogate model developed in this study. The first direction involves investigating a broader range of scenarios (e.g., launch parameters). In this study we have constructed and analysed an surrogate model for the *conservative* scenario described in Section 3.4. However, as discussed in that section, several scenarios can be envisioned that each will lead to a different projected evolution of the space debris environment. Using the high fidelity simulator developed in this study, it is conceptually easy (but computationally demanding) to construct new surrogate models for these different scenarios. However, when computational power is available, the methodology we developed will make it easy to run the required Monte Carlo simulations to build a new model, which can then be analysed in the same fashion as we have done in this study for the conservative scenario.

The second extension concerns the addition of mega constellations to the simulator, in addition to the three classes of satellites we have considered in this paper (see Section 3.3.2). Mega constellations are currently being considered as a new addition to traditional satellite operation. Besides adding significant numbers of satellites to the space environment, mega constellations would constantly replenish their supply of satellites over a long period of time. If left unmitigated, this has been shown to have a profound effect on the space environment (Rossi et al., 2017) and is thus worth including in the simulation.

## Author Contributions

All authors contributed to the conception and design of the experiments. RK throughout performed the experiments and analysed the data. RK, DB, RS and KT contributed to the game theoretic analysis. AW, AS and DI provided input on space debris, active debris removal and the launch model. All authors wrote the paper.

## Conflict of Interest Statement

The authors declare that the research was conducted in the absence of any commercial or financial relationships that could be construed as a potential conflict of interest.

## Footnotes

NORAD tracks and catalogues objects in orbit, currently listing around 15,000 objects of 10 cm^2^ and larger, see https://celestrak.com/NORAD/elements/. It is believed that the true number of objects is several orders of magnitude larger, with estimates of over 100,000 pieces of un-tracked debris of sizes 1-10 cm^2^ (Carrico et al., 2008).

https://github.com/richardklima/Space_debris_removal_dynamic

https://celestrak.com

https://www.space-track.org/

https://github.com/dnwrnr/sgp4

run on i7-2600 CPU @ 3.40GHz, 16GB of RAM

In this paper we use the term Stochastic game due to the common usage in the literature. However we assume deterministic transitions and Deterministic game would be more accurate.

In this paper we will use the term reward even for negative values, which can be thought of as a cost.
